# sTREM-1 promotes the phagocytic function of microglia to induce hippocampus damage via the PI3K–AKT signaling pathway

**DOI:** 10.1038/s41598-022-10973-8

**Published:** 2022-04-29

**Authors:** Li Lu, Xuan Liu, Juanhua Fu, Jun Liang, Yayi Hou, Huan Dou

**Affiliations:** 1 The State Key Laboratory of Pharmaceutical Biotechnology, Division of Immunology, Medical School, Nanjing University, Nanjing, 210093 People’s Republic of China; 2 Jiangsu Key Laboratory of Molecular Medicine, Nanjing, 210093 People’s Republic of China; 3 Department of Rheumatology and Immunology, Nanjing Drum Tower Hospital, The Affiliated Hospital of Nanjing University Medical School, Nanjing, 210008 People’s Republic of China

**Keywords:** Immunology, Molecular biology

## Abstract

Soluble triggering receptor expressed on myeloid cells-1 (sTREM-1) is a soluble form of TREM-1 released during inflammation. Elevated sTREM-1 levels have been found in neuropsychiatric systemic lupus erythematosus (NPSLE) patients; yet, the exact mechanisms remain unclear. This study investigated the role of sTREM-1 in brain damage and its underlying mechanism. The sTREM-1 recombinant protein (2.5 μg/3 μL) was injected into the lateral ventricle of C57BL/6 female mice. After intracerebroventricular (ICV) injection, the damage in hippocampal neurons increased, and the loss of neuronal synapses and activation of microglia increased compared to the control mice (treated with saline). In vitro. after sTREM-1 stimulation, the apoptosis of BV2 cells decreased, the polarization of BV2 cells shifted to the M1 phenotype, the phagocytic function of BV2 cells significantly improved, while the PI3K–AKT signal pathway was activated in vivo and in vitro. PI3K–AKT pathway inhibitor LY294002 reversed the excessive activation and phagocytosis of microglia caused by sTREM-1 in vivo and in vitro, which in turn improved the hippocampus damage. These results indicated that sTREM-1 activated the microglial by the PI3K–AKT signal pathway, and promoted its excessive phagocytosis of the neuronal synapse, thus inducing hippocampal damage. sTREM-1 might be a potential target for inducing brain lesions.

## Introduction

Systemic lupus erythematosus (SLE) is a chronic autoimmune disease that can affect any organ system in the body, causing widespread inflammation and tissue damage in the affected organs^1^. According to epidemiological studies on SLE, the prevalence of SLE in women is significantly higher than that in men, particularly those in their childbearing age^2–4^. More than 50% of SLE patients develop significant psychiatric and neurologic manifestations, also known as neuropsychiatric SLE (NPSLE), which severely affect the life quality of patients and increase disease-related mortality^5^. Accumulating evidence suggested that complex and interconnected mechanisms promote the development of SLE-related brain damage; yet, the underlying mechanism of pathogenesis is still not well understood. Neuroinflammation is believed to be one of the major pathogenetic mechanisms in NPSLE^6^. Therefore, identifying the key assumptions in brain inflammation and damage may contribute to alleviating NPSLE.

Triggering receptor expressed on myeloid cells-1 (TREM-1) is a transmembrane protein expressed on neutrophils, monocytes, and microglia. TREM-1 is an amplifier of the innate immune response during various inflammatory diseases^7–9^. The soluble form of TREM-1 (sTREM-1) is produced by cleaving the extracellular ectodomain of membrane-bound TREM-1 or mRNA alternate spicing and exists as a soluble monomer^10–15^. Since sTREM-1 retains the extracellular domain of membrane-bound TREM-1, it can act as a decoy receptor, which competitively binds to the receptor of TREM-1 and weakens its pro-inflammatory activity^16^. High concentrations of sTREM-1 have been found in multiple infectious and chronic inflammatory diseases such as pneumonia, acute myocardial infarction, sepsis, familial Mediterranean fever, Type 1 diabetes, inflammatory bowel diseases, rheumatoid inflammatory disorders, and cancer^17–25^. Thus, it is believed that sTREM-1 may be a marker of TREM-1 activation and potentially a biomarker for diagnosing inflammatory diseases.

Three studies reported that increased serum sTREM-1 levels are associated with disease activity and infection^26–28^. Moreover, higher plasma levels of sTREM-1 have been detected in subgroups of patients with neuropsychiatric manifestations, particularly those with the total high disease activity score and NPSLE activity^27^. In addition, increased sTREM-1 levels have been found in the cerebral spinal fluid (CSF) of subarachnoid hemorrhage patients, monitoring the extent of brain injury at an early stage^29,30^. Also, after acute ischemic stroke, the serum levels of sTREM-1 in patients are positively correlated with S100B, a marker for brain damage and blood–brain-barrier disruption^31^. Yet, the exact function of sTREM-1 in NPSLE remains unclear.

Microglia is the key effector and regulator in the central nervous system (CNS), which participates in almost all brain diseases^32^. As the unique mononuclear macrophages in the CNS, microglia maintain the homeostasis of the brain environment by eliminating pathogens and dead cells^32–34^. When the brain is in a pathological state, microglia enter an “activated state” characterized by shortening and swelling of the cellular processes, changes in the surface phenotypes and secretory mediators, and increased proliferation response^35^. On the one hand, activated microglia promote the body's inflammatory response and then change the brain's synaptic plasticity and cognitive deficits, thereby inducing brain diseases^36^. On the other hand, in addition to phagocytosis of dead cells, activated microglia immoderately engulf viable cells, and lead to normal cell death, subsequently exacerbating brain injury or disturbing normal development^37^. Previous reports have shown that microglia activation promotes overly engulf synapses, leading to a greater loss of neurons in Alzheimer's disease (AD), Parkinson's Disease (PD), and ischemic stroke^38–42^. Such neuronal loss culminates in an increased brain tissue loss and exacerbated long-term functional deficits^43^. Moreover, excessive microglial phagocytosis of synapses is also considered to be the cause of various neurological diseases such as schizophrenia^44,45^, tauopathy^46,47^, and multiple sclerosis (MS)^48,49^. Therefore, activated microglia are a common pathological feature of a series of neurodegenerative diseases^50–52^.

Microglia have been proven to be an important contributor to NPSLE^53^. Abnormal microglial activation has been detected in the hippocampus of several strains of lupus-prone mice (NZB/NZW, MRL/lpr, and FcγRIIB-/-Yaa)^54^. In the process of continuous inflammation, activated microglia-phagocyted astrocytes promote neuronal apoptosis and aggravate depression index and cognitive dysfunction in lupus mice^55–57^. Nestor et al*.* demonstrated activated microglia could induce neuronal damage in NPSLE mice^58^. However, it is still not clear whether sTREM-1 directly mediates microglial dysfunction and participates in brain injury.

In this study, we examined the role of sTREM-1 in the severity of brain damage and microglia functional outcomes. To investigate the effects of high levels of sTREM-1 in the brain, sTREM-1 recombinant protein was intracerebroventricular injected into the lateral ventricle of mice. Then hippocampal neurons damage and synapse density were immediately measured at different time points. The underlying mechanisms were also assessed in vitro and in vivo.

## Materials and methods

### Mouse model

Specific pathogen-free female C57BL/6 mice (aged 6–8-weeks, weighting 20 ± 2 g) were purchased from the Model Animal Research Center of Nanjing University (Nanjing, Jiangsu Province, China). All mice were maintained under specific pathogen-free conditions at a 12 h light/dark cycle and 20–22 °C. The animals were allowed free access to drinking water. The experiments on mice were approved by Institutional Animal Care and Use Committee, Nanjing University, and all experiments were performed in accordance with relevant guidelines and regulations. This study was carried out in compliance with the ARRIVE guidelines.

**Figure 1:**
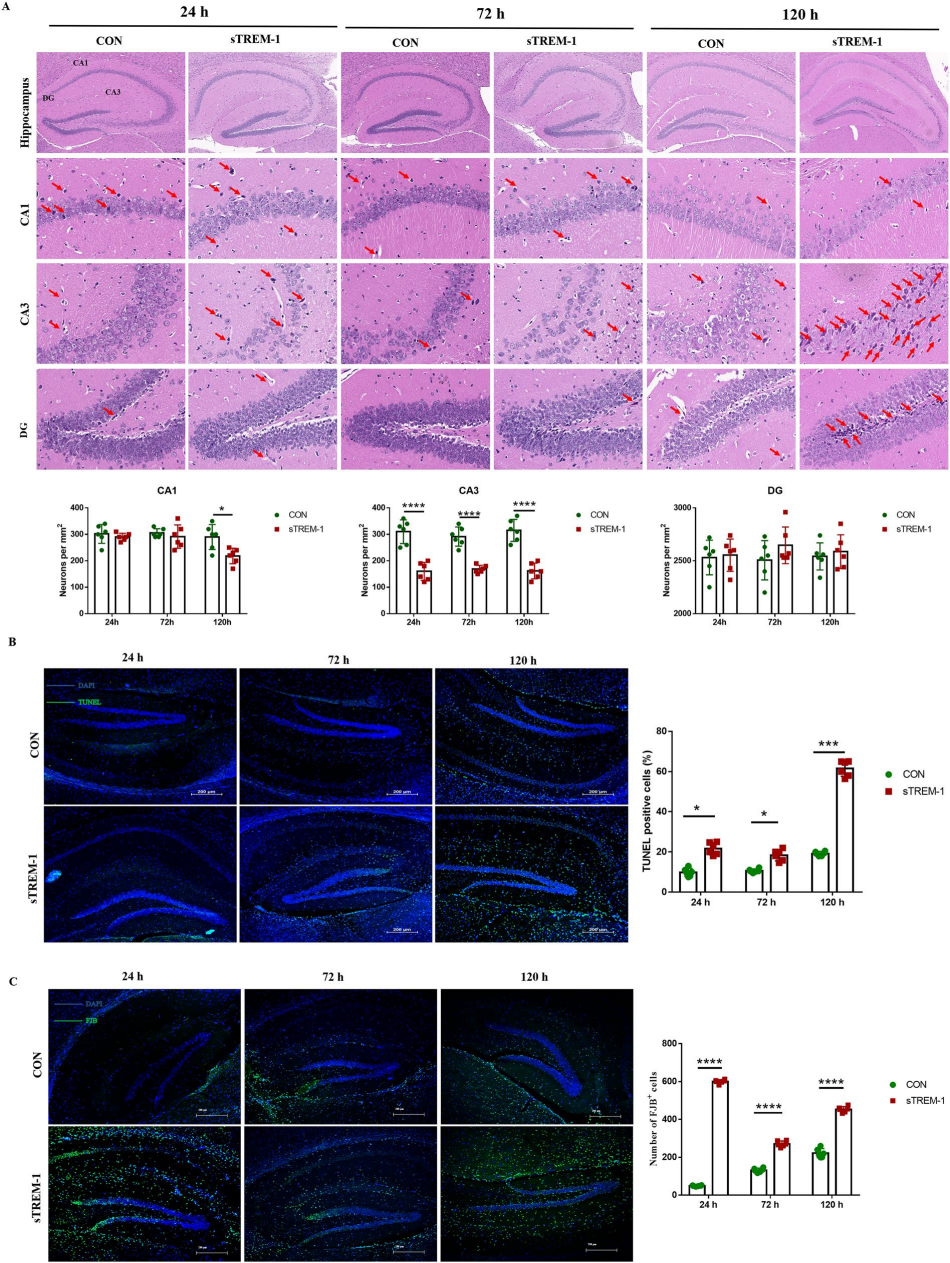
sTREM-1 induces hippocampal neurons damage in mice of mice. **(A)** H&E staining was used to observe the morphological changes of neurons in the CA1, CA3, and DG areas of the mouse hippocampus, n = 6. **(B)** Representative fluorescence image of hippocampal TUNEL staining of mice; scale bar is 200 μm; and the statistical analysis of the percentage of TUNEL positive cells in the entire hippocampus, n = 6. **(C)** Representative fluorescent images of hippocampal FJB staining of mice, scale bar is 200 μm; and the number of FJB positive cells in the entire hippocampus, n = 6. Data are presented as means ± SEM of at least three separate experiments, *P < 0.05, ***P < 0.001, ****P < 0.0001.

Firstly, the experimental process for the effect of sTREM-1 on brain function is shown in Fig. 1A. A total of 90 female C57BL/6 mice were divided into six groups: (i) 24 h-CON group (n = 15), (ii) 24 h-sTREM-1 group (n = 15), (iii) 72 h-CON group (n = 15), (iv) 72 h- sTREM-1 group (n = 15), (v) 120 h-CON group (n = 15), (vi) 120 h- sTREM-1 group (n = 15). For mice in the sTREM-1-treated groups, sTREM-1 (Sino Biological Inc. Beijing, China) recombinant protein (2.5 μg) at a volume of 3 μL was injected into the right lateral ventricle of the mice brain. The CON group mice were treated with the equivalent volume of vehicle (saline). Bregma served as the origin of coordinates, the intracerebroventricular (ICV) injection was performed perpendicular to the skull (x = 0.8 mm, y = 0.2 mm, z = 2 mm) using a microprocessor-controlled mini-pump^59^, delivery was performed at a rate of 500 nL/min. After injection, the needle was left in place for 5 min prior to slowly retracting it from the ventricles^59^. Afterward, the mice were left underneath the warm light to recover their mobility. The mice were then sacrificed at the corresponding time.

Secondly, the experimental process for evaluation of PI3K–AKT signaling in sTREM-1-mediated brain injury is shown in Fig. 7A. A total of 30 female C57BL/6 mice were divided into three groups: (i) CON group (n = 10), (ii) sTREM-1 group (n = 10), (iii) sTREM-1 + LY294002 group (n = 10). PI3K–AKT pathway inhibitor LY294002 (MedChemExpress, USA) was diluted with phosphate-buffered saline (PBS) to 10 mM, each mouse received an injection of 3 µL. Fifteen minutes before the ICV injection of sTREM-1, LY294002 was injected into the mouse brain. Similarly, the CON group used saline as a control. After sTREM-1 treatment for 120 h, the mice were sacrificed for the next study.

### Hematoxylin/eosin (H&E) staining

Six mice per group were used for Hematoxylin/eosin (H&E) staining. The mice were cardiac perfused with cold 4% paraformaldehyde immediately after being anesthetized with pentobarbital sodium (50 mg/kg ip) (Sigma-Aldrich, Germany). The whole brain of the mice was fixed in 4% paraformaldehyde for 48 h before being embedded for paraffin sectioning. Series of coronal sections (5 μm) were taken containing cortex and hippocampus behind the bregma (Bregma −1.6 to −2.1 mm)^60,61^, then these sections were used for hematoxylin/eosin (H&E) staining.

### Immunofluorescence detection

Brain tissue sections were performed as previously described, and in accordance with the manufacturer’s protocol (Merck & Millipore Company, Germany), the FluoroJade B (FJB) staining was carried out.

Immunofluorescence staining was performed as previously described^62^, the paraffin sections were soaked in cold acetone for 15 min. After washing with PBS, pre-cooled methanol was added dropwise and treated at −20 °C for 30 min. Then it was blocked with 3% BSA for 60 min at room temperature. The TUNEL (Roche, Switzerland) reaction mixture or CD68 (Boster, Wuhan, China)/IBA-1 (Abways Technology, Shanghai, China) primary antibodies were added at room temperature and incubated overnight at 4 °C, then the corresponding secondary antibodies were added for binding, followed by nuclei staining with DAPI. The slides were visualized using a Nikon Eclipse Ti-U fluorescence confocal microscope, which was equipped with a digital camera (FV300, Olympus, Japan).

### Extraction of total RNA and quantitative real-time PCR

Half of the left and right hippocampus of four mice per group were used for quantitative real-time PCR (qRT-PCR) detection. Total RNA was extracted from cells or tissues using TRIzol reagent. According to the manufacturer’s instructions, 1 μg total RNA was reverse transcribed in a 20-μL reaction system. The oligonucleotide primers used for PCR amplification were listed in Table 1. All reactions were carried out in triplicate. The mRNA levels were normalized to that of *β-actin*. The primers were synthesized by Springen (Nanjing, China). The 5′-3′ primer sequences were as follows:

**Table 1:** Primers used for q-PCR in this study.

Gene	Forward	Reverse
*β-actin*	GGCTGTATTCCCCTCCATCG	CCAGTTGGTAACAATGCCATGT
*Dlg4*	GGCGGAGAGGAACTTGTCC	AGAATTGGCCTTGAGGGAGGA
*SYN1*	CCAATCTGCCGAATGGGTACA	GCGTTAGACAGCGACGAGAA
*Homer1*	GTTCTCAGCCCAACAATACAAGA	GTGGACGGGTCGATGTCAC
*PI3K*	GCAGAGGGCTACCAGTACAGA	CTGAATCCAAGTGCCACTAAGG
*AKT*	ATGAACGACGTAGCCATTGTG	TTGTAGCCAATAAAGGTGCCAT

### Western blotting

Half of the left and right hippocampus of four mice per group were used for western blot detection. Western blot was performed as previously described^62^. Briefly, hippocampus tissues or cells were lysed in ice-cold RIPA lysis buffer containing protease and phosphatase inhibitors. Supernatants were used for SDS-PAGE and western blots. An equivalent amount of 30 μg protein was separated by SDS-PAGE, transferred to a 0.22 μm polyvinylidene fluoride (PVDF) membrane. The membranes were blocked in 5% BSA for 2 h at room temperature. The membranes were treated with specific primary antibodies reacting to GAPDH (Goodhere Technology, Cat. No. AB-P-R 001), β-actin (Bioworld, Cat. No. BS607M), PSD95, SYN1, and Homer1 (Proteintech, Cat. No. 20665-1-AP, 20258-1-AP, and 12433-1-AP, respectively), Bcl-XL, caspase-3, cleaved-caspase-3, phospho-PI3K, PI3K, phospho-AKT and AKT (Cell Signaling Technology, Cat. No. 2764, 9662, 9661, 4228, 4257, 4060, 4691, respectively), Bcl-2 (Bioworld, Cat. No. BS1511). Followed by incubation with appropriate HRP-linked secondary antibody for 2 h at room temperature. The immunoreactive bands were visualized using enhanced Chemiluminescence (ECL) plus western blot detection reagents (SupersignalTM West Pico PLUS, Thermo, USA). The grey level of proteins was calculated by the Image J software. In the experiment, GAPDH or β-actin was used as a protein loading control.

### Flow cytometric analysis

Five mice per group were used for flow cytometric analysis. Perform sample preparation and flow cytometry according to the previous experimental description^62^. Briefly, after immunomagnetic beads sorting, single cells were pre-blocked with anti-CD16/CD32 Fc Blocker for 10 min, then stained with Zombie NIR™ Fixable Viability Kit (Biolegend, CA, USA), CD11b-FITC (clone number: M1/70, BD Biosciences, CA, USA), CD45-APC (clone number: RA3-6B2, Biolegend, CA, USA) antibodies for 30 min at 4 °C in the dark. Rinsed twice with PBS, and finally re-suspended in 200 μL buffer for subsequent evaluation by flow cytometry (CYTEKTM NL-2000, China). FlowJo software (TreeStar, Ashland, OR, USA) was applied for data analysis. Flow cytometry is used to distinguish between resting and activated microglia in the brain based on the expression intensity of CD45 and CD11b. The resting microglia is labeled as CD45^low^CD11b^+^, and the activated microglia is defined as CD45^int^CD11b^+^^63–65^.

### Cell culture and viability analysis

BV2 cells were generous gifts from Prof. Tianjiao Xia, Nanjing University, and were cultured in DMEM containing 10% FBS at 37 °C with 5% CO2. In the in vitro experiment, BV2 cells were treated with sTREM-1 (0.1 μg/ml and 1 μg/ml) for 6 h, or use LY294002 (10 μM) pretreatment for 1 h in advance.

For the CCK8 experiment, BV2 cells (1 × 10^4^ cells/well) were seeded in 96-well plates to assess cell viability. Following overnight culture, cells were incubated with various concentrations of sTREM-1 (0.00625–25.6 μg/ml) for 24 h. Subsequently, 10 μL of CCK-8 reagent (Fcmacs, China) was added to each well and incubated for an additional 0.5–4 h. The absorbance at 450 nm was measured by a microplate reader (Bio Tek, Winooski, VT, USA).

The xCELLigence Real-Time Cell Analysis (RTCA) instrument (ACEA Biosciences, Hangzhou, China) was used to detect the proliferation of BV2 cells in real-time. Cells were harvested and diluted to a seeding density of 1 × 10^4^ cells/well and were seeded to the E-plate in 100 µL volume. The cell index was monitored every 5 min by the RTCA DP analyzer.

### RNA-seq and bioinformatic data analysis

BV2 cells were seeded into a six-well plate at a density of 2 × 10^5^ cells/well and cultured overnight (n = 4). Then, BV2 cells were incubated with sTREM-1 recombinant protein (1ug/mL) for 6 h. Total RNA from BV2 cells was extracted using TRIzol reagent (Vazyme Biotech, China). All analytical samples were sent to NovelBio (Shanghai NovelBio Bio-Pharm Technology Co., Ltd) for the RNA sequence assay. Briefly, total ribonucleic acid (RNA) from BV2 cells was extracted using Trizol reagent according to the manufacturer's instructions. The total RNA that had a standard of concentration ≥ 200 ng/μL, mass ≥ 10 μg, and RNA integrity number (RIN) ≥ 8.0 was subjected to RNA-Seq. Raw reads were pre-processed using FastQC software. Transcript expression levels were estimated using fragments per kilobase per million reads (FPKM) values and quantified by RSEM software. EdgeR software was used to identify differentially expressed genes. For functional enrichment analysis, all DEGs were mapped to terms in the GO databases, and then significantly enriched GO terms were searched for among the DEGs using *P* < 0.05 as the threshold. GO term analysis was classified into three subgroups, namely biological process (BP), cellular component (CC), and molecular function (MF). All DEGs were mapped to the KEGG database and searched for significantly enriched KEGG pathways at *P* < 0.05 level^66^. The datasets generated during and/or analyzed during the current study are available in the GEO repository with identifier GSE185830.

### Microglial phagocytosis detection

The phagocytic capacity of microglia was determined using the method described previously^67^. BV2 cells were seeded into a 24-well plate at a density of 5 × 10^4^ cells/well and cultured overnight. Pre-incubate FBS and 1-µm amine-modified polystyrene latex beads at 37 °C for 1 h at a ratio of 1:5, and then added the mixture to DMEM medium to ensure that the ratio of latex beads is 0.01%. After incubating the DMEM medium containing latex beads (Miltenyi Biotec, Germany) with the treated BV2 cells for 2 h. The number of fluorescent beads phagocytosed by BV2 could be detected by flow cytometry.

We co-cultured BV2 cells with HT22 cells to detect the change of phagocytosis on sTREM-1 treated BV2 cells. The BV2 cells and HT22 cells were co-cultured at a ratio of 1:3 for 24 h. Afterward, samples were collected for the corresponding testing.

### Statistical analysis

All data are presented as mean ± standard error of the mean (SEM), and each experiment included triplicate sets. Comparison between two means was done by Student’s t-test. Comparison between three or more means was done by one-way analysis of variance (ANOVA) with a Tukey’s multiple comparisons test. *P* < 0.05 was considered statistically significant. GraphPad Prism 7 was used for data analysis (GraphPad Software Inc., CA, USA).

### Ethical approval

All animal studies were approved by Institutional Animal Care and Use Committee, Nanjing University, and all experiments were performed in accordance with relevant guidelines and regulations.

## Results

### sTREM-1 induces hippocampal neurons damage in mice

In order to clarify the role of sTREM-1 infiltrating the brain, we injected sTREM-1 recombinant into the mice’s brains. After 24, 72, and 120 h post-injection, H&E staining was used to observe the neuronal morphology of the mice hippocampus. Compared with the control group, the number of neurons in the CA3 area was significantly reduced, and the cell structure was blurry after 24 h. After 72 h, a small amount of irregularly shaped neuronal cells appeared in the CA3 and DG areas. After 120 h, the neurons in the CA1 and CA3 areas were disorderly arranged, and their number was significantly reduced; irregular-shaped neuronal cells increased, and a large number of ghost cells appeared in the hippocampal DG area (Fig. 1A). These results indicated that sTREM-1 can directly destroy neuronal cell morphology and induce severe neuronal degeneration in a time-dependent manner.

In order to clarify the effect of sTREM-1 on hippocampal damage, we labeled the apoptotic cells in the mouse hippocampus by TUNEL staining (Fig. 1B). Compared with the CON group mice, the proportion of apoptotic cells in the hippocampus significantly increased in a time-dependent manner (Fig. 1B) (*P* < 0.05). At 120 h, the number of apoptotic cells in the mice hippocampus of the sTREM-1 group increased nearly 3 times compared to the control mice (Fig. 1B) (*P* < 0.001). In addition, the damaged neurons in the hippocampus by FJB staining showed that the number of FJB-positive dying neurons in the sTREM-1 group was significantly increased compared with the CON group (Fig. 1C) (*P* < 0.0001). These results suggested that sTREM-1 promotes hippocampal cells apoptosis and neurons damage.

### sTREM-1 reduces the expression of synaptic markers in the hippocampus of mice

**Figure 2:**
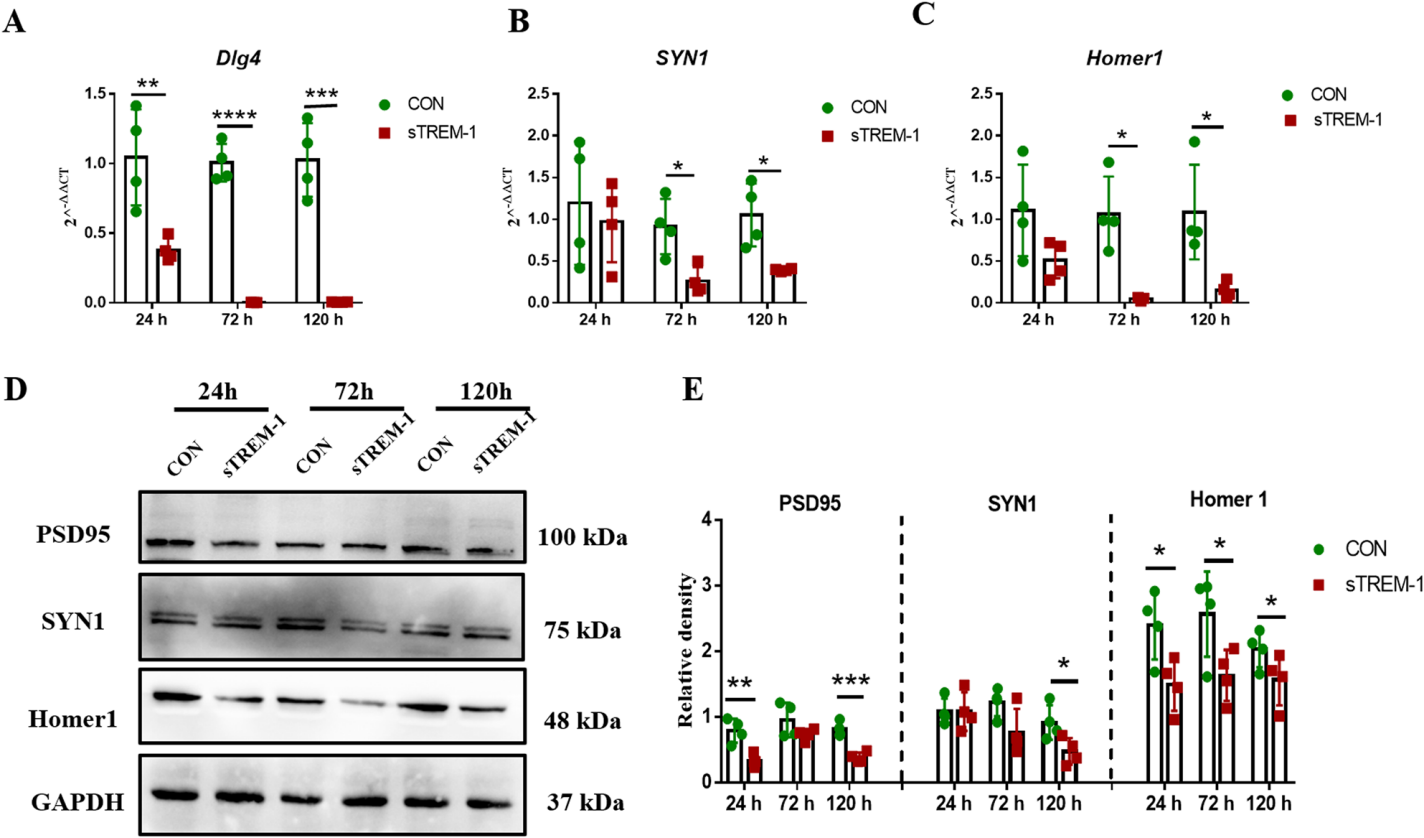
sTREM-1 reduces the expression of synaptic markers in the hippocampus of mice. **(A)** The mRNA expression levels of *Dlg4* in mouse hippocampus detected by Q-PCR, n = 4. **(B)** The mRNA expression levels of *SYN1* in mouse hippocampus detected by Q-PCR, n = 4. **(C)** The mRNA expression levels of *Homer1* in mouse hippocampus detected by Q-PCR, n = 4. **(D)** Representative western blot images of PSD95, SYN1, and Homer1 protein expression in mouse hippocampus, n = 4. **(E)** Quantitative analysis of PSD95, SYN1 and Homer1 protein level in mouse hippocampus, n = 4. Data are presented as means ± SEM of at least three separate experiments, *P < 0.05, **P < 0.01, ***P < 0.001, ****P < 0.0001.

Phagocytosis is an important part of the innate immune response, which has a defensive role against pathogens during the infection process^68,69^. However, the excessive activation of microglia phagocytosis leads to synapses damage, thereby promoting neuronal degeneration^70,71^. Thus, we detected the expression of synaptic markers in the hippocampus of the CON and sTREM-1 groups. Compared with the CON group, the mRNA expression of *Dlg4* was down-regulated in a time-depended manner (Fig. 2A) (*P* < 0.01). Similarly, the protein PSD95 encoded by Dlg4 was also reduced (Fig. 2D,E). At the same time, the gene and protein levels of postsynaptic membrane markers SYN1 and Homer1 were similarly down-regulated (Fig. 2B–D,E) (*P* < 0.05). These results indicated that after ICV injection of sTREM-1, neuronal synapses were lost in the hippocampus of mice.

### sTREM-1 promotes the activation of microglia in vivo and in vitro

**Figure 3:**
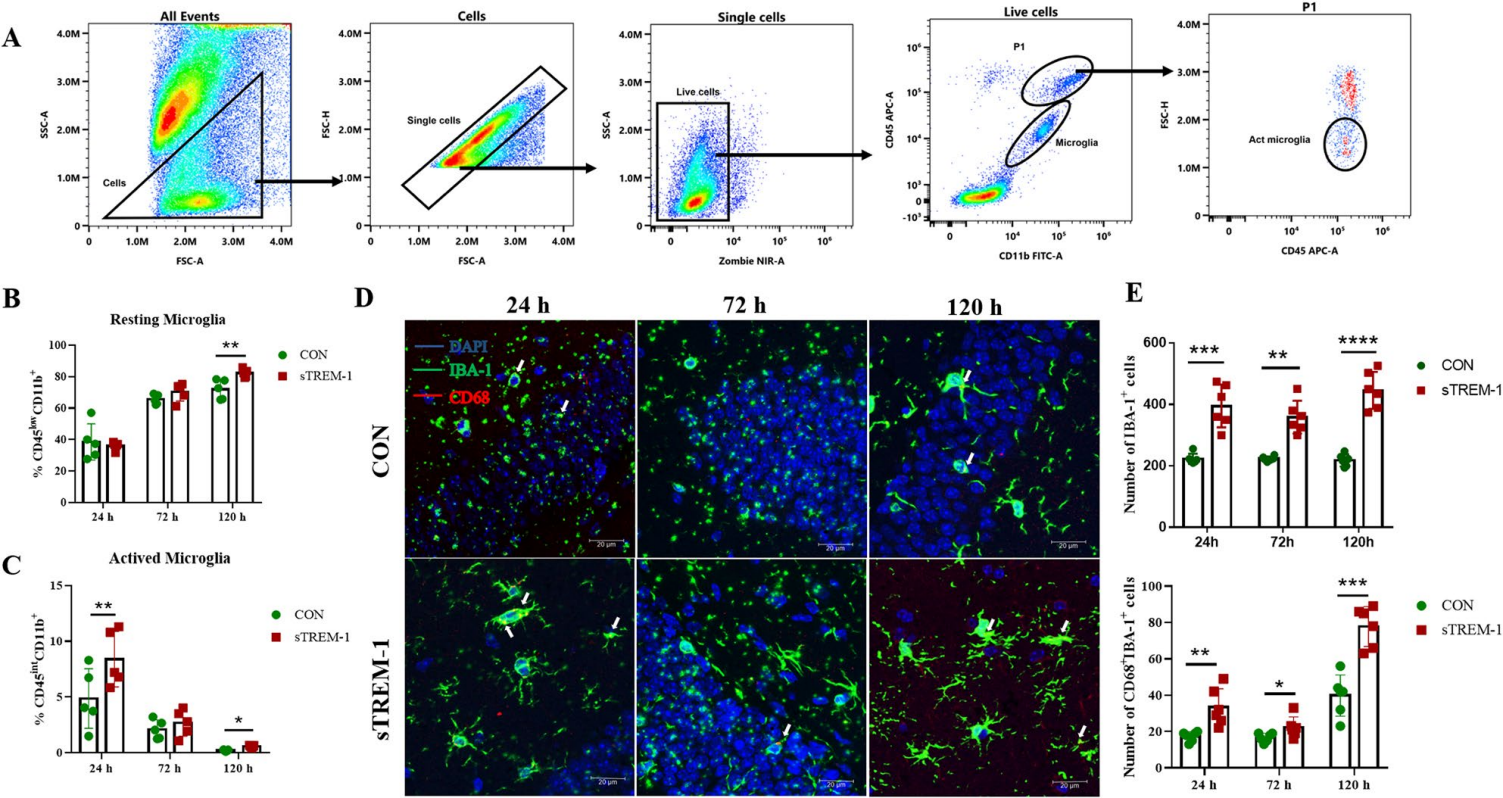
sTREM-1 promotes the activation of microglia in the hippocampus of mice. **(A)** The gating strategy of resting and activating microglia in the mouse hippocampus. **(B)** Quantitative analysis of the proportion of resting microglia in the hippocampus of per group mice, n = 5. **(C)** Quantitative analysis of the proportion of activated microglia in the hippocampus of each group mice, n = 4. **(D)** Representative image of IBA-1 and CD68 immunofluorescence double staining of the hippocampus in per group mice, where the white arrows indicated CD68^+^IBA-1^+^ cells, scale bar is 200 μm. **(E)** Quantitative analysis of IBA-1^+^, IBA-1 and CD68 double-positive cells, n = 6; Data are presented as means ± SEM of at least three separate experiments, *P < 0.05, **P < 0.01, ***P < 0.001, ****P < 0.0001.

Microglia can be present in a resting state and an activated state. Under pathological conditions, resting microglia are activated, their branching increases and their phagocytosis is enhanced^72^. In order to clarify whether the synaptic loss was related to the enhanced phagocytosis of microglia, we detected the number and phenotypic changes of microglia in the hippocampus by flow cytometry (Fig. 3A). Compared with the 24 h-CON groups, the proportion of resting microglia in the 24 h-sTREM-1 group did not change, while the activated microglia significantly increased (Fig. 3B,C) (*P* < 0.05). However, in the 72 h-CON and 72 h-sTREM-1 groups, there were no differences in the proportion of resting and activated microglia (Fig. 3B,C). Contrary, in the 120 h-sTREM-1 group, both resting and activated microglia significantly increased (Fig. 3B,C) (*P* < 0.05).

In order to visualize the morphology of microglia, IBA-1 and CD68 immunofluorescence staining were used to analyze the morphology and activation status of microglia. The results showed that compared with the CON group, the total and branched microglia and the number of IBA-1^+^CD68^+^ cells in the sTREM-1 group significantly increased (Fig. 3D,E) (*P* < 0.05). These results indicated that sTREM-1 treatment increased the number of microglia in the hippocampus and promoted its polarization towards a pro-inflammatory phenotype.

**Figure 4:**
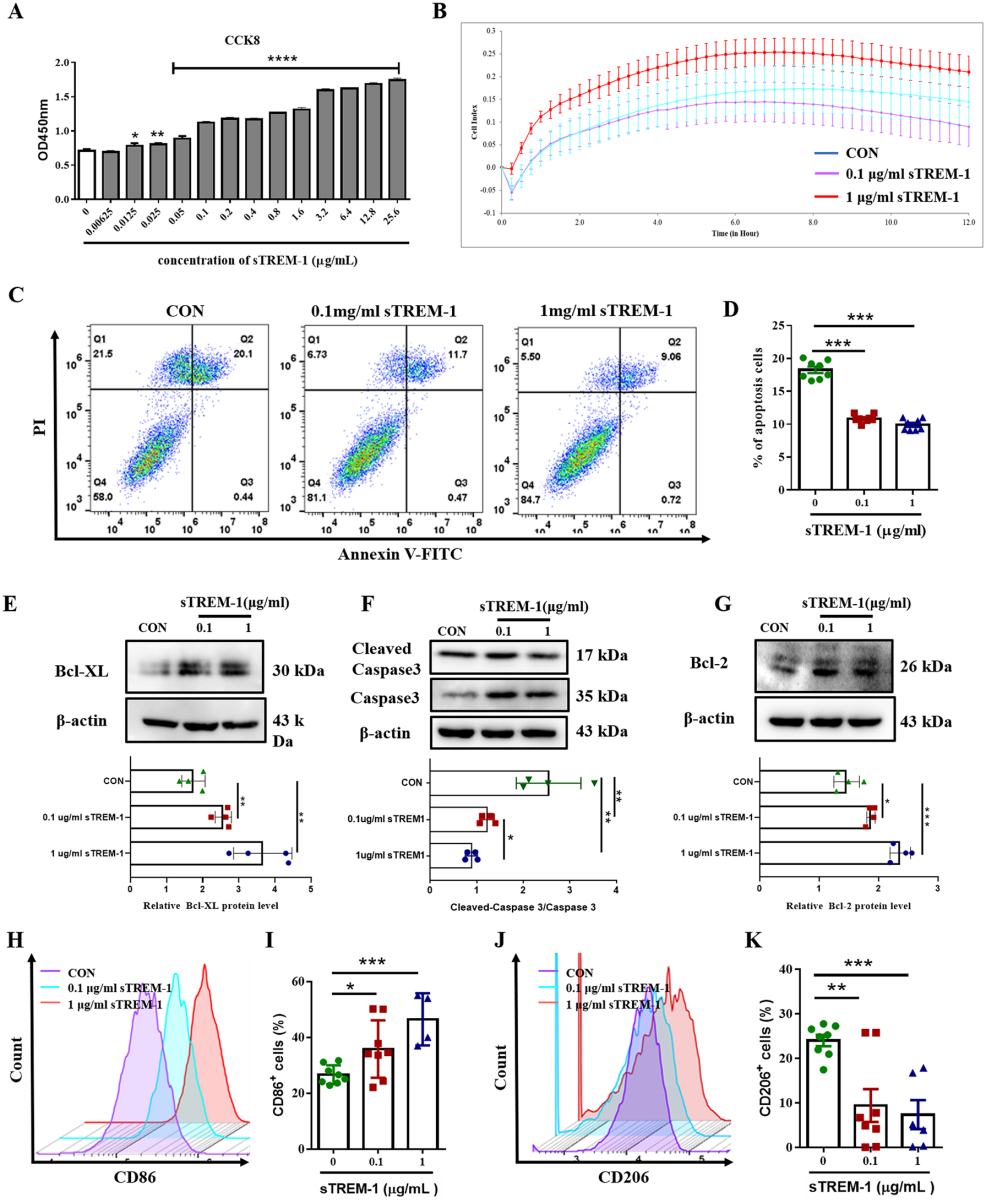
sTREM-1 promotes the activation of BV2 cells in vivo*.* **(A)** CCK-8 was used to detect the cytotoxicity of sTREM-1 on BV2 cells. **(B)** RTCA was used to detect the effect of sTREM-1 on the proliferation of BV2 cells in each treatment group. **(C)** Representative images of the apoptotic levels of BV2 cells in each treatment group were detected by flow cytometry. **(D)** The percentage of early apoptotic (Annexin V^+^PI^+^) cells in each treatment group. **(E)** Representative western blot images and quantitative analysis of Bcl-XL protein expression levels of BV2 cells in each treatment group. **(F)** Representative western blot images and quantitative analysis of caspase-3 protein expression levels of BV2 cells in each treatment group. **(G)** Representative western blot images and quantitative analysis of Bcl-2 protein expression levels of BV2 cells in each treatment group. **(H)** Representative images of M1 phenotype (CD86^+^) in BV2 cells by flow cytometry detection. **(I)** Quantitative analysis of the proportion of CD86^+^ BV2 cells in each treatment group. **(J)** Representative images of M1 phenotype (CD206^+^) in BV2 cells by flow cytometry detection**. (K)** Quantitative analysis of the proportion of CD206^+^ BV2 cells in each treatment group; Data are presented as means ± SEM of at least three separate experiments. *P < 0.05, **P < 0.01, ***P < 0.001, ****P < 0.0001.

Next, we treated BV2 cells with sTREM-1 to explore the regulation of microglia in vitro. First, CCK-8 was applied to seek a safe dose. The results showed that sTREM-1 promotes BV2 cells proliferation in a dose-dependent manner (Fig. 4A). Next, we selected 0.1 μg/mL and 1 μg/mL for follow-up experiments. Besides, RTCA also confirmed the proliferative effect of sTREM-1 on BV2 cells (Fig. 4B), which was consistent with the results of animal experiments. Flow cytometry showed that late apoptotic cells (Annexin V^+^PI^+^) were reduced after sTREM-1 treatment (Fig. 4C,D) (*P* < 0.001). Similarly, the western blot result showed that sTREM-1 up-regulated the expression of anti-apoptotic proteins Bcl-2, Bcl-XL, and down-regulated the expression of pro-apoptosis protein caspase-3 (Fig. 4E–G), which indicated that sTREM-1 reduces the apoptosis of BV2 cells. At the same time, we found that sTREM-1 intensified the differentiation of BV2 cells to the M1 phenotype and weakened their differentiation to the M2 phenotype (Fig. 4H–K). All the above results suggested that sTREM-1 promoted the activation of microglia in vivo and in vitro.

### sTREM-1 enhances the phagocytosis of microglia

**Figure 5:**
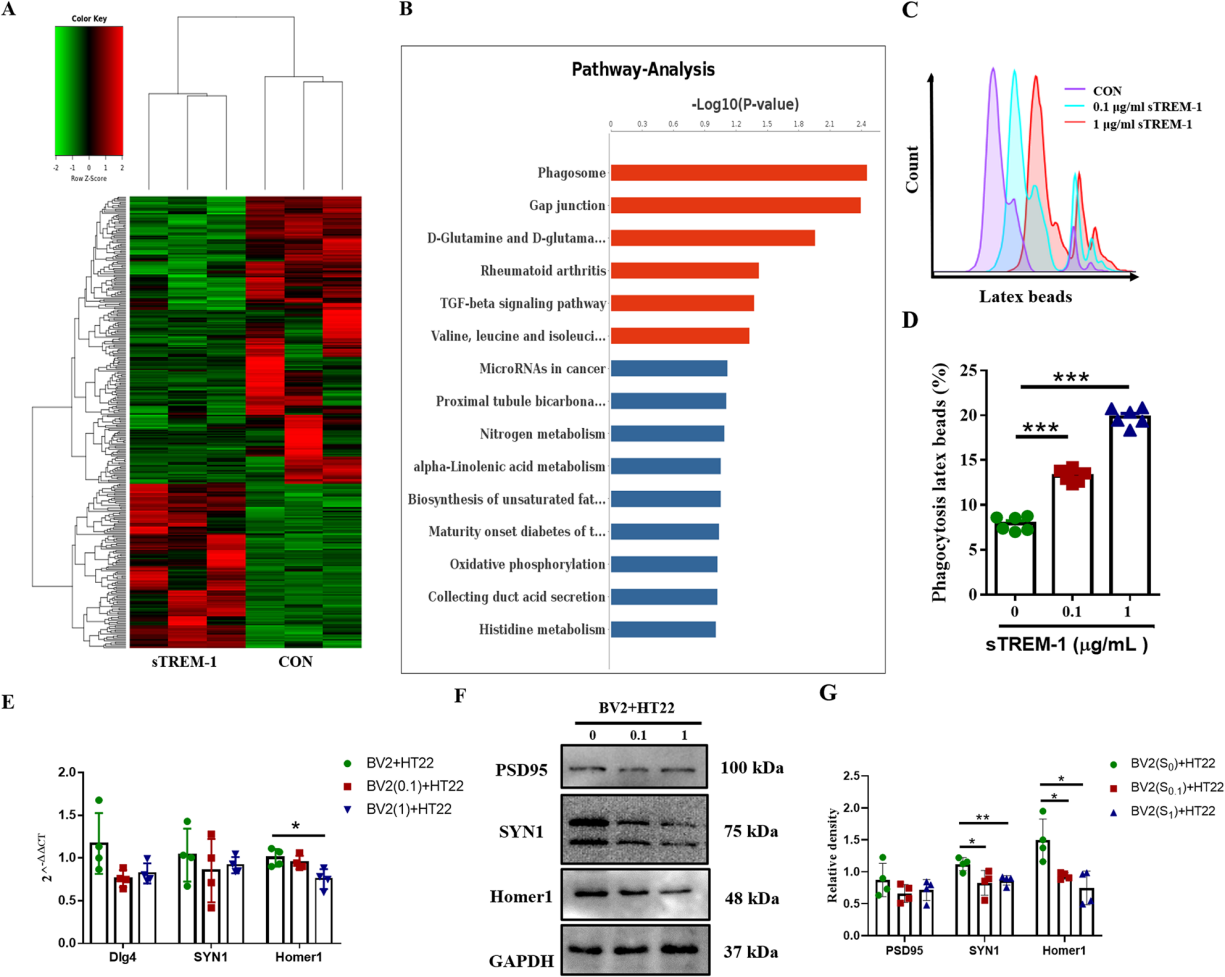
sTREM-1 enhances the phagocytosis of BV2 cells. **(A)** A heat map of all RNAs detected in the BV2 cells treated with or without sTREM-1 by transcriptomics sequencing. Red indicates upregulation; green indicates downregulation. **(B)** Pathway-Analysis diagram of BV2 cells in CON group and sTREM-1 treatment group. **(C)** Representative histograms of BV2 cells phagocytosis of latex beads were detected by flow cytometry in different treatment groups. **(D)** After BV2 cells were treated with sTREM-1, a phagocytic function test was performed, and the phagocytosis of BV2 cells was analyzed using flow cytometry. **(E)** The mRNA expression levels of *Dlg4*, *SYN1*, and *Homer1* in different HT22 cell groups by Q-PCR. **(F)** Representative western blot images of PSD95, SYN1, and Homer1 protein expression in different HT22 cell groups. **(G)** Quantitative analysis of Dlg4, SYN1, and Homer1 protein levels in different HT22 cell groups. Data are presented as means ± SEM of at least three separate experiments. *P < 0.05, **P < 0.01, ***P < 0.001.

In order to clarify the regulation of sTREM-1 on microglial biological functions, we stimulated BV2 cells with sTREM-1 (1ug/mL) by RNA-seq; untreated BV2 cells were used as a control (Fig. 5A). Pathway-Analysis results showed that the phagocytosis of BV2 cells was significantly up-regulated after sTREM-1 stimulation (about 2.4 times, Fig. 5B). The latex bead phagocytosis experiment further verified this phenomenon; after sTREM-1 treatment, the phagocytic efficiency of fluorescent beads by BV2 cells increased nearly three times (Fig. 5C,D) (*P* < 0.001). In addition, we co-cultured BV2 cells (treated with sTREM-1) with HT22 cells for 24 h to observe the phagocytosis of BV2 cells on synapses of HT22 cells. The results showed that the expression of the synaptic marker Homer1 on HT22 cells was significantly down-regulated after co-cultivation with sTREM-1-treated BV2 cells (Fig. 5E–G), while the protein level of SYN1 was down-regulated (Fig. 5F,G) (*P* < 0.05). These results indicated that sTREM-1 enhances the phagocytosis of BV2 cells.

### sTREM-1 activates the PI3K-AKT pathway in vivo and in vitro

**Figure 6:**
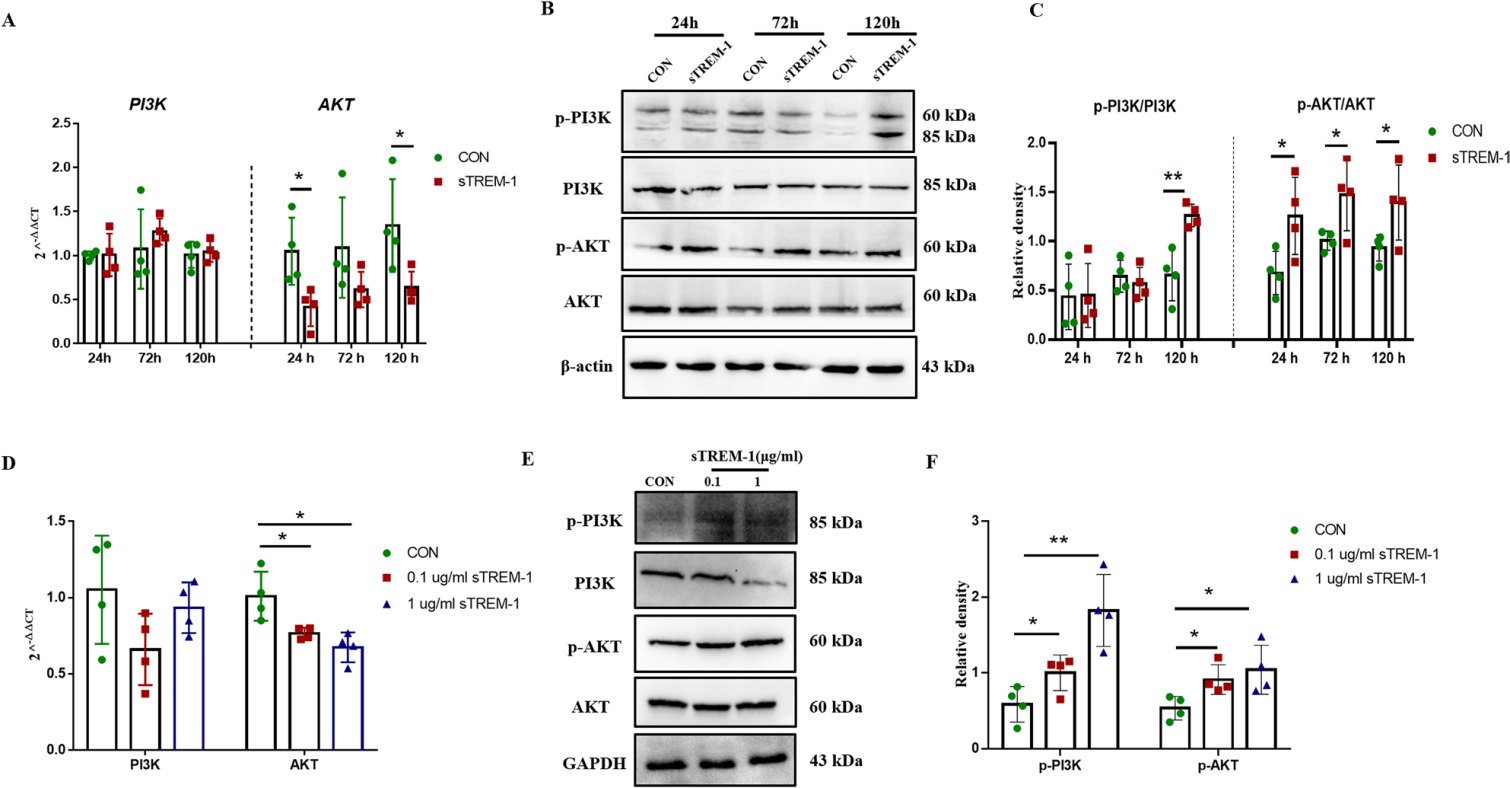
sTREM-1 activates the PI3K-AKT pathway in vivo and in vitro. **(A)** The mRNA expression levels of *PI3K* and *AKT* in mouse hippocampus detected by Q-PCR, n = 4. **(B)** Representative western blot images of p-PI3K, PI3K, p-AKT and AKT protein expression in mouse hippocampus. **(C)** Quantitative analysis of p-PI3K/PI3K and p-AKT/AKT protein level in mouse hippocampus, n = 4. **(D)** The mRNA expression levels of *PI3K* and *AKT* in different BV2 cells groups by Q-PCR, n = 4. **(E)** Representative western blot images of p-PI3K, PI3K, p-AKT, and AKT protein expression in different BV2 cell groups. **(F)** Quantitative analysis of p-PI3K/PI3K and p-AKT/AKT protein level in different BV2 cell groups, n = 4. Data are presented as means ± SEM of at least three separate experiments. *P < 0.05, **P < 0.01.

A PI3K–AKT signaling pathway is a key pathway for TREM-1, TREM-2, and sTREM-2 to participate in brain injury^73–75^. However, the relationship between the sTREM-1 and PI3K–AKT signaling pathway has not yet been reported. In this study, we firstly examined the expression of PI3K and AKT in the hippocampus of mice and found that the mRNA level of *AKT* was significantly reduced after sTREM-1 treatment compared with the CON group (Fig. 6A); yet, no difference was found in *PI3K* gene expression (Fig. 6A). At the same time, the phosphorylation of AKT (p-AKT) protein significantly increased at different periods (Fig. 6B,C), and the expression of phosphorylated PI3K (p-PI3K) protein was up-regulated in the 120 h-sTREM-1 group (Fig. 6B,C). These results suggested that sTREM-1 induced hippocampal damage might be related to the activation of the PI3K-AKT pathway.

In order to clarify whether the influence of sTREM-1 on the PI3K-AKT signaling pathway was related to microglia, we stimulated BV2 cells with sTREM-1 to detect the expression of PI3K and AKT. Compared with the CON group, sTREM-1 treatment reduced the mRNA expression of *AKT* but showed no effect on *PI3K* (Fig. 6D). After sTREM-1 treatment, the levels of p-AKT and p-PI3K protein significantly increased (Fig. 6E,F); the above results were highly consistent with the situation in the hippocampus. Accordingly, we verified the activation of phagocytosis (phagocytosis of fluorescent microbeads and neuronal cells) in the primary microglia cells after sTREM-1 recombinant protein or LY294002 treatment and obtained similar results (Fig. S1A–C). These results suggested that sTREM-1 induces hippocampal through the activation of the PI3K–AKT pathway in microglia.

### Inhibition of the PI3K-AKT pathway reduces the phagocytic activation of microglia induced by sTREM-1

**Figure 7:**
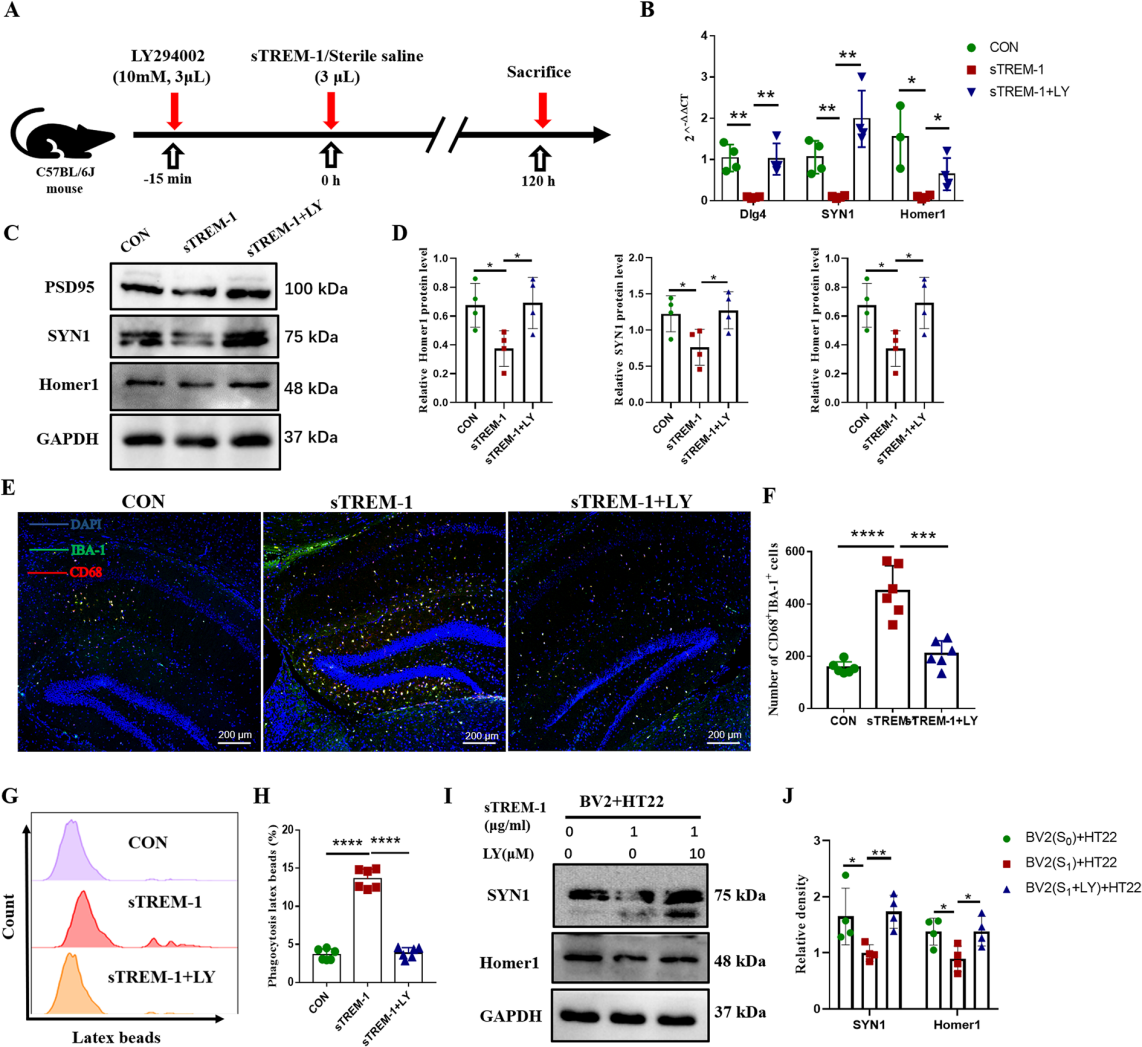
Inhibition of the PI3K–AKT pathway attenuates the phagocytic activation of microglia induced by sTREM-1. **(A)** The experimental plan was visualized. **(B)** The mRNA expression levels of *Dlg4*, *SYN1,* and *Homer1* in mouse hippocampus detected by Q-PCR, n = 4. **(C)** Representative western blot images of Dlg4, SYN1, and Homer1 protein expression in mouse hippocampus. **(D)** Quantitative analysis of PSD95, SYN1, and Homer1 protein level in mouse hippocampus, n = 4. **(E)** Representative image of IBA-1 and CD68 immunofluorescence double staining of the hippocampus in per group mice, scale bar is 200 μm. **(F)** Quantitative analysis of IBA-1 and CD68 double-positive cells, n = 6. **(G)** Representative histograms of BV2 cells phagocytosis of latex beads in different treatment groups detected by flow cytometry. **(H)** Phagocytosis in different BV2 cells groups detected by flow cytometry. **(I)** Representative western blot images of SYN1 and Homer1 protein expression in different HT22 cells groups. **(J)** Quantitative analysis of SYN1 and Homer1 protein level in different HT22 cells groups. Data are presented as means ± SEM of at least three separate experiments, *P < 0.05, **P < 0.01, ***P < 0.001, ****P < 0.0001.

In order to clarify whether sTREM-1 regulates the phagocytosis of microglia through the PI3K-AKT signaling pathway, we injected PI3K-AKT inhibitor LY294002 15 min before sTREM-1 injection (Fig. 7A). LY294002 significantly suppressed the expression of p-AKT in the hippocampus (Figures S2A-C). Subsequently, we detected the expression of synaptic markers in the mouse hippocampus and found that inhibition of the PI3K-AKT signaling pathway reversed the down-regulation of synaptic markers after sTREM-1 treatment. After LY294002 treatment, the expression of synaptic markers PSD95, SYN1, and Homer1 significantly increased (Fig. 7B–D).

Immunofluorescence staining was used to detect the co-localization of CD68 and IBA-1 in the hippocampus. The increase of CD68^+^IBA-1^+^ cells mediated by sTREM-1 was significantly reduced after the PI3K-AKT pathway inhibition (Fig. 7E,F).

Next, we pretreated BV2 cells with LY294002 and detected the effect of sTREM-1 on phagocytosis in vitro. The results showed that LY294002 pretreatment significantly inhibited the phosphorylation of AKT (Fig. S2D–F). We also found that the phagocytosis of BV2 cells could not be activated by sTREM-1 after pre-treatment with LY294002 (Fig. 7G,H). In addition, the BV2 cells and HT22 cells co-cultivation experiment also proved that the PI3K-AKT pathway was inhibited in advance; the phagocytosis of BV2 cells could not be overactivated by sTREM-1 (Fig. 7I,J). These results suggested that sTREM-1 induced the activation of microglia phagocytosis depending on the PI3K-AKT pathway in vitro.

### Inhibition of PI3K-AKT signaling pathway improves sTREM-1 induced hippocampal injury in mice

**Figure 8:**
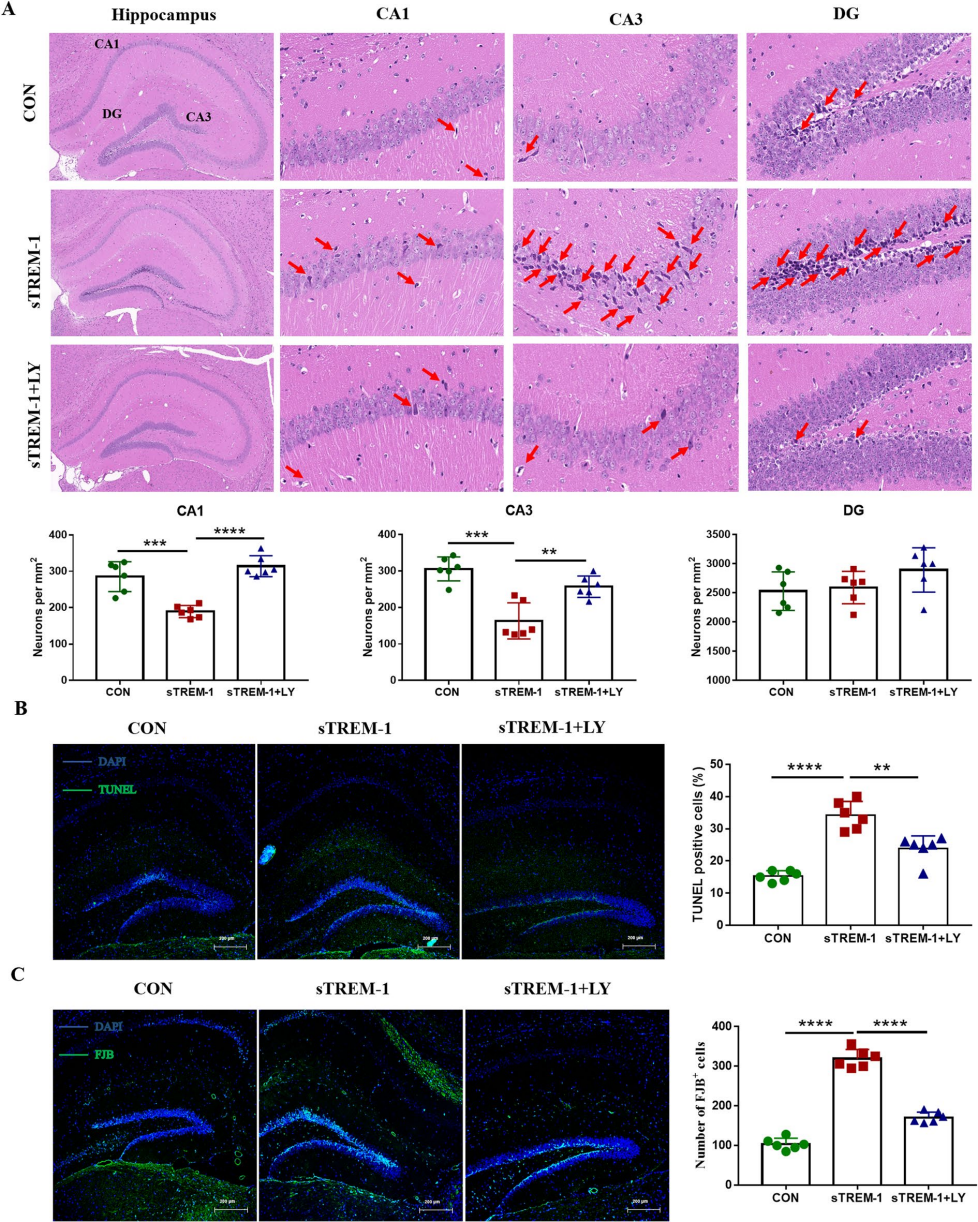
Inhibition of PI3K-AKT signaling pathway improves sTREM-1 induced hippocampal injury in mice. **(A)** H&E staining was used to observe the morphological changes of neurons in the CA1, CA3, and DG areas of the mouse hippocampus, n = 6. **(B)** Representative fluorescence image of hippocampal TUNEL staining of mice; scale bar is 200 μm; and the percentage of TUNEL positive cells in the entire hippocampus, n = 6. **(C)** Representative fluorescent images of hippocampal FJB staining of mice; scale bar is 200 μm; and the number of FJB positive cells in the entire hippocampus, n = 6. Data are presented as means ± SEM of at least three separate experiments, **P < 0.01, ***P < 0.001, ****P < 0.0001.

The regulation of brain function and brain development is related to the PI3K–AKT signaling pathway^76–78^. We speculated that sTREM-1 induces brain damage through the activation of the PI3K–AKT pathway. Therefore, after inhibiting the PI3K–AKT pathway, we observed the effect of sTREM-1 on the hippocampus. Compared with the CON group, the number of neurons in the CA1 and CA3 regions of the hippocampus decreased, the structures were blurry, and the ghost cells in the CA3 and DG regions increased in the sTREM-1 group. After pre-treatment with LY294002, the number of neurons was rebound to a certain extent, the morphology of neurons was restored, and the number of ghost cells reduces (Fig. 8A). In addition, we also detected the damage of total cells and neurons in the hippocampus. TUNEL and FJB staining results indicated that the inhibition of the PI3K–AKT signaling pathway could attenuate the total cell and neuronal injury induced by sTREM-1 (Fig. 8B,C). These results suggested that sTREM-1 induced hippocampal damage through the activation of the PI3K–AKT pathway.

## Discussion

This study explored the potential effect of sTREM-1 on hippocampus damage and its molecular regulatory mechanism. Our results indicated that sTREM-1 recombinant protein enhances the phagocytosis of microglia by activating the PI3K–AKT signaling pathway, thus further inducing hippocampal damage. These data suggest that sTREM-1 might be a potential molecular target to regulate brain damage.

sTREM-1, a 27 kDa protein composed of the extracellular domain of mTREM-1, is present in various body fluids and was initially identified as a biomarker of infectious diseases^79–84^. Later on, it was discovered that plasma sTREM-1 participates in neurodegeneration and brain injury. In patients with AD, plasma sTREM-1 is significantly increased and has been associated with disease progression, dementia, and total Tau levels^85^. Xin-Gang Sun et al. reported that the increase of sTREM-1 is positively related to the severity of early brain injury induced by subarachnoid hemorrhage^29^, while its inhibition can impede the activation of microglia, reduce the destruction of the blood–brain barrier and neuronal damage, and improve short-term and long-term neurological function^86–88^.

Serum sTREM-1 has been considered a surrogate index for evaluating the severity and functional prognosis of patients with acute ischemic stroke^31^. A previous study indicated an increased TREM-1 in serum, spleen, and lymph nodes of B6.lpr mice. Apart from this, increased serum sTREM-1 was observed in patients with increased NPSLE compared with healthy subjects and was positively correlated with the severity of the disease^46^. In addition, the levels of sTREM-1 in the serum, brain tissue, and cerebrospinal fluid of NPSLE mice were also increased^27^. In this study, we injected sTREM-1 recombinant protein into the right lateral ventricle of mice and found altered neurons morphology and increased neurons death, thus suggesting that sTREM-1 may induce hippocampal neurons damage.

As an indispensable part of the brain's innate immune system, resident microglia can quickly respond to brain damage and neurological diseases^89^. On the one hand, microglia are activated after injury to participate in the regulation of neuronal damage and brain function^90^; on the other hand, as professional phagocytes, microglia regulate brain development through superior clearance effects^91^. Our results showed that sTREM-1 reduces the expression of synaptic markers and up-regulates the population of resting and activated microglia. Studies have shown that "activated microglia" are usually transformed from "resting microglia" in order to protect the CNS from neuronal damage and the subsequent neuroinflammatory response^92^. However, once microglia are over-activated, the inflammatory process is enhanced, leading to synaptic function and plasticity loss, cognitive deficits, and in turn, the occurrence of brain diseases, such as MS, AD, and PD^36^. Hence, activated microglia are considered to be a common pathological feature of neurodegenerative diseases^50–52^. Our results showed that hippocampal microglia were polarized towards the M1 (CD86^+^) pro-inflammatory phenotype, suggesting the activation of the phagocytic function of microglia^37,93^. Cell experiments further showed that sTREM-1 stimulation promoted proliferation and enhances the phagocytic function of BV2 cells.

Studies have shown that mTREM-1 is the positive regulator of immune cell phagocytosis. Tao et al. reported that mTREM-1 enhances the microglial phagocytosis in AD^94^. Inhibition of mTREM-1 in RAW264.7 cells can reduce its phagocytosis of lipids, while its activation enhances the ability of polymorphonuclear neutrophils to swallow latex beads^95^. sTREM-1 is a special form of TREM-1 that can be directly tested in human body fluids. Previous studies have shown that it serves as a decoy receptor in multiple models of sepsis, attenuating inflammation and markedly improving survival^7,96,97^. Nevertheless, recent extensive studies have found that sTREM-1 is a reliable biomarker for the diagnosis of infectious diseases, particularly in septic shock, and specifically indicates the TREM-1 pathway activation^98–100^. In respiratory diseases, sTREM-1 impairs the innate immune response and facilitates the development of serious secondary bacterial infections^14^. The above results suggest that the function of sTREM-1 is still not entirely clear. Although there is no clear report on whether the abnormal expression of sTREM-1 in the CNS is related to the excessive activation of microglia, TREM2, a member of the same family with TREM-1, is selectively expressed in microglia and is considered as a receptor for microglia phagocytosis, participating in the regulation of CNS homeostasis^101,102^. The activation of TREM-2 is involved in the anti-inflammatory process of microglia and may promote their phagocytosis^38,102,103^. Patients with TREM2-mutations have severe neurological sequelae^104^. Meanwhile, sTREM-2 appears to induce inflammation of microglia, and enhance their phagocytic function^73,105^. However, the effect of sTREM-1 on phagocytosis is still poorly understood. Our results indicated that sTREM-1 enhanced the phagocytic function of microglia, which filled the gap in the field.

Phosphatidylinositol 3-kinase (PI3K)-Serine/threonine kinase (AKT) pathway participates in the regulation of brain metabolism and synapse formation^76,78,106^. The activation of the PI3K–AKT pathway promotes the transfer of nuclear factor-κB (NF-κB) into the nucleus, thereby inducing inflammatory factors, aggravating the deterioration of the brain environment, and destroying the synaptic plasticity in the brain^77^. The activation of the PI3K–AKT signaling pathway is an important inducement of brain malformations^78^. If the PI3K–AKT signaling pathway is inhibited, inflammation can be alleviated, and brain damage caused by cold stress can be prevented^106^. In addition, the role of the PI3K–AKT signaling pathway in neurodegenerative diseases has also been widely explored^107^. Glycogen synthase kinase-3 beta (GSK-3β) in AD patients has a negative feedback regulation relationship with the PI3K-AKT signaling pathway. Activation of GSK-3β blocks the PI3K-AKT pathway in neural stem cells and increases the hyperphosphorylation of Tau protein to induce neurotoxicity^108,109^. Furthermore, the PI3K–AKT pathway is a key pathway for multiple members of the TREM family to exert a regulatory role. TREM-1 induces inflammation and lipid accumulation in human hepatoma cell line HepG2 and primary mouse hepatocytes by activating the PI3K-AKT pathway^74^. TREM-2 promotes the differentiation of osteoclasts, increases the production of inflammatory factors, and enhances the killing effect of bacteria in the murine macrophage cell line RAW264.7 through the PI3K–AKT pathway^75,110,111^. sTREM-2 is dependent on the PI3K–AKT pathway to induce inflammation of microglia, promoting their survival and enhancing its phagocytic function in vivo and in vitro^73,105^. In our experiment, we found that sTREM-1 activated the PI3K-AKT pathway in the mouse hippocampus and BV2 cells. Other studies have reported that the activation of the PI3K-AKT pathway is required for microglia to exert their phagocytic activity^112,113^. In order to investigate whether sTREM-1 mediates microglial activation and brain damage by activating the PI3K–AKT signaling pathway, we first inhabited the PI3K–AKT pathway before treating cells with sTREM-1. The results showed that inhibited PI3K–AKT signaling pathway could reduce the activation of microglial phagocytosis and brain damage mediated by sTREM-1.

Our study first reported that sTREM-1 enhances microglial phagocytosis by activating the PI3K–AKT pathway, after which excessive phagocytosis of the neuronal synapse induces hippocampal damage. This research indicated that sTREM-1 could be a potential therapeutic target for brain injury.

## Supplementary Information

Supplementary Figures.

Supplementary Figures.

## Data Availability

The datasets generated during and/or analyzed during the current study are available in the GEO repository with the identifier GSE185830.

## Abbreviations

SLE: Systemic lupus erythematosus
NPSLE: Neuropsychiatric systemic lupus erythematosus
AD: Alzheimer’s disease
PD: Parkinson's disease
MS: Multiple sclerosis
CNS: Central nervous system
FJB: Fluoro-Jade B
HE: Hematoxylin–eosin stain
Homer1: Homer scaffold protein 1
mTREM-1: Membrane-bound triggering receptor expressed on myeloid cells -1
sTREM-1: Soluble triggering receptor expressed on myeloid cells -1
PFA: Paraformaldehyde
GAPDH: Glyceraldehyde-3-phosphate dehydrogenase
PI3K: Phosphatidylinositol 3 kinase
PSD95: Postsynaptic density protein 95
SYN1: Synapsin I
