# Supplementary material for: sTREM-1 promotes the phagocytic function of microglia to induce hippocampus damage via the PI3K–AKT signaling pathway
Source: Sci Rep. 2022 Apr 29;12:7047. doi: 10.1038/s41598-022-10973-8 (PMC9054830; doi:10.1038/s41598-022-10973-8)
Supplement: Supplementary file 1 — Supplementary Figures. [file 41598_2022_10973_MOESM1_ESM.pdf]

sTREM-1 promotes the phagocytic function of microglia to induce hippocampus damage via the PI3K-AKT signaling pathway

Li Lu[1, 2#], Xuan Liu[1, 2#], Juanhua Fu[1, 2#], Yayi Hou[1,2*], Huan Dou[1,2*]

1. The State Key Laboratory of Pharmaceutical Biotechnology, Division of Immunology, Medical School, Nanjing University, Nanjing 210093, PR China

2. Jiangsu Key Laboratory of Molecular Medicine, Nanjing, 210093, PR China, 210093

3. Department of Rheumatology and Immunology, Nanjing Drum Tower Hospital, The Affiliated Hospital of Nanjing University Medical School, Nanjing 210008, PR China.

[#] These authors contributed equally to this work.

Corresponding author: Huan Dou, Email: douhuan@nju.edu.cn; Yayi Hou, Email: yayihou@nju.edu.cn; Jun Liang, Email: 13505193169@139.com.

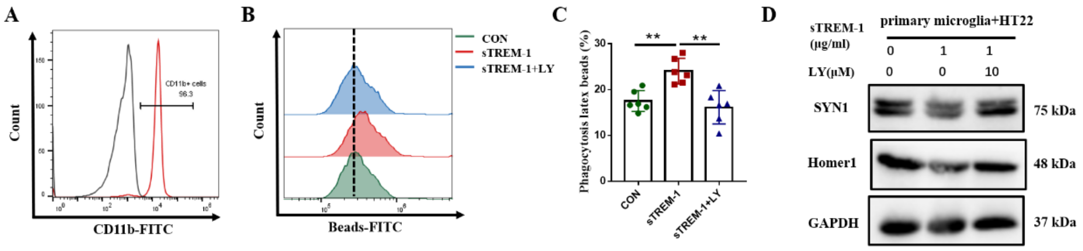

Supplementary Fig. 1 sTREM-1 enhances the phagocytosis of primary microglia by activating the PI3K-AKT pathway.

(A) Validation of the purity of primary microglia obtained by flow cytometry; **(B)** After primary microglia were treated with sTREM-1 (2 μg/mL), a phagocytic function test was performed, and the phagocytosis of primary microglia was analyzed using flow cytometry. Representative histograms of primary microglia phagocytosis of latex beads detected by flow cytometry in different treatment groups; **(C)** Quantitative analysis of Figure B; **(D)** Representative western blot images of SYN1 and Homer1 protein expression in different HT22 cell groups. Data are presented as means ± SEM of at least three separate experiments. $^{**}P < 0.01$.

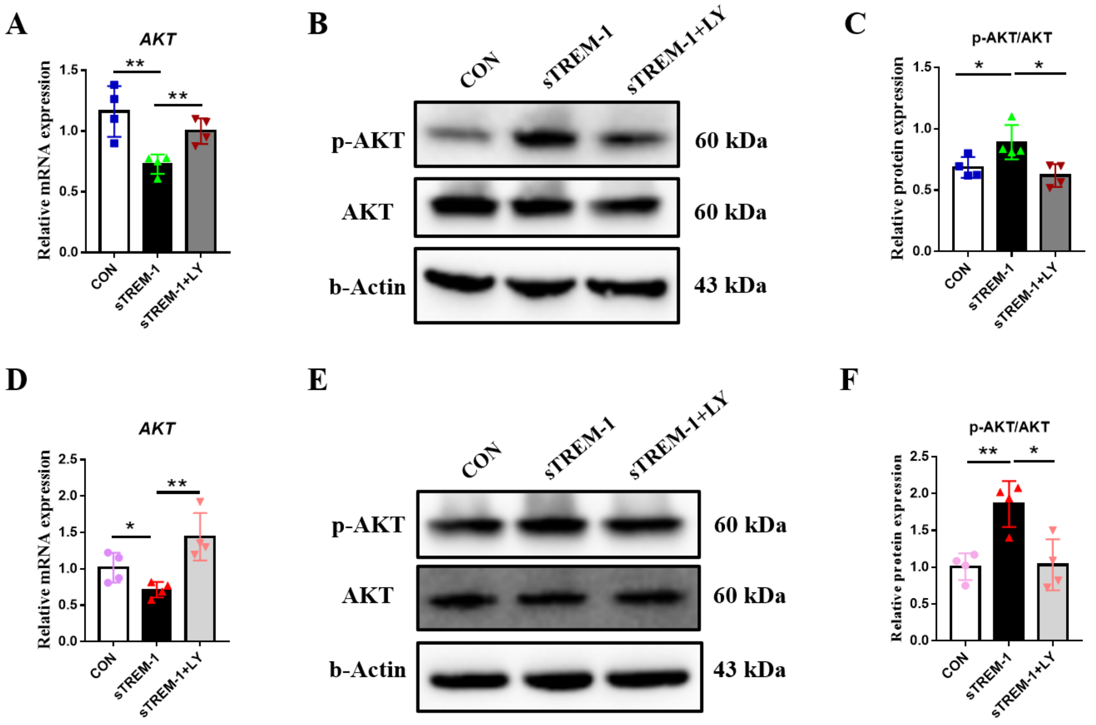

Supplementary Fig. 2 LY294002 inhibits phosphorylation of AKT in vivo and in vitro.

(A) The mRNA expression levels of AKT in mouse hippocampus was detected by Q-PCR, n=4; **(B)** Representative western blot images of p-AKT and AKT protein expression in mouse hippocampus; **(C)** Quantitative analysis of p-AKT/AKT protein level in the mouse hippocampus, n=4; **(D)** The mRNA expression levels of AKT in BV2 cells was detected by Q-PCR, n=4; **(E)** Representative western blot images of p-AKT and AKT protein expression in BV2 cells; **(F)** Quantitative analysis of p-AKT/AKT protein level in BV2 cells, n=4; Data are presented as means ± SEM of at least three separate experiments. *$P < 0.05$, **$P < 0.01$.
