# Supplementary material for: sTREM-1 promotes the phagocytic function of microglia to induce hippocampus damage via the PI3K–AKT signaling pathway
Source: Sci Rep. 2022 Apr 29;12:7047. doi: 10.1038/s41598-022-10973-8 (PMC9054830; doi:10.1038/s41598-022-10973-8)

Supplemental Figure 1. Full Western Blot Images for Figure 2 D.

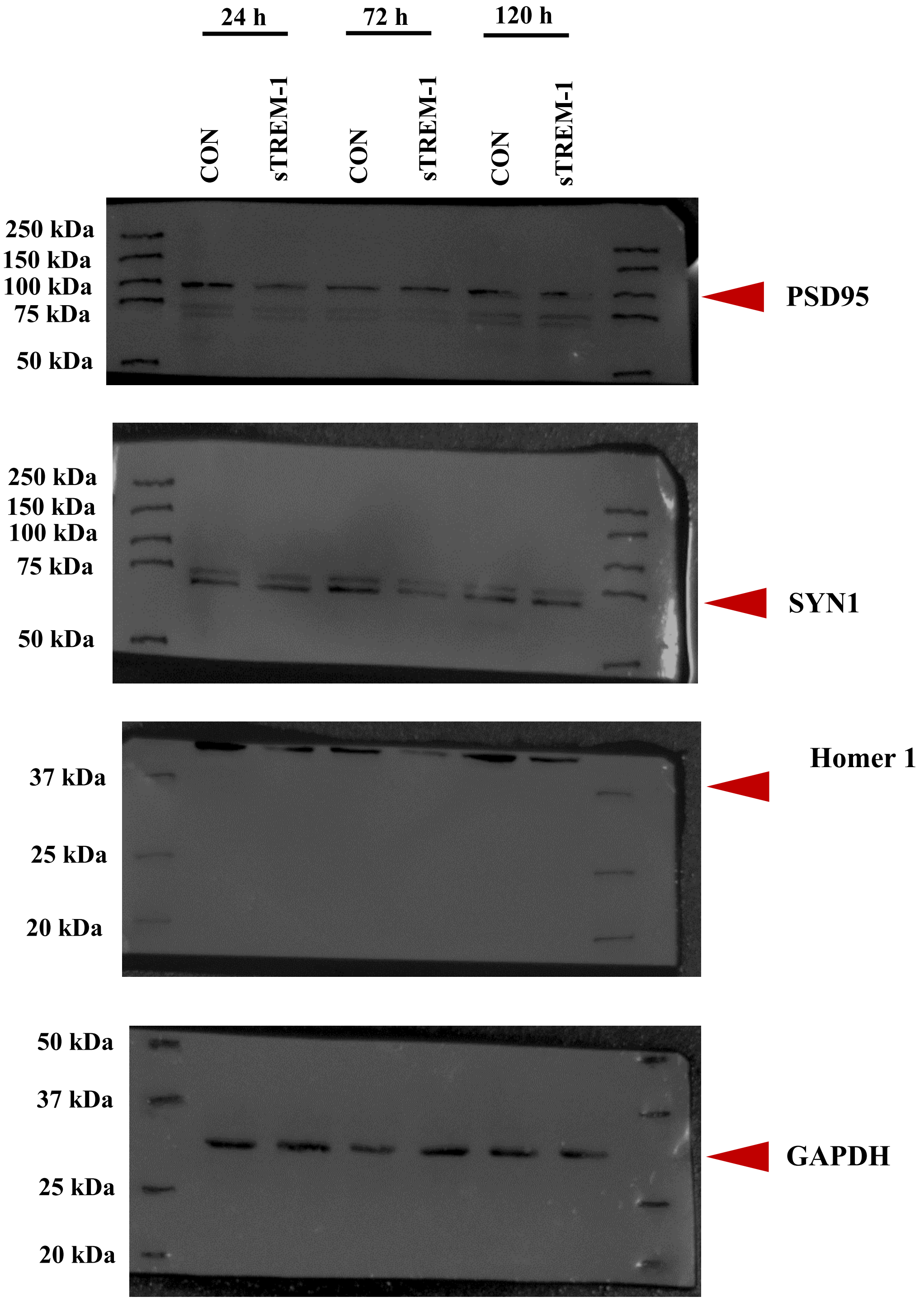

Supplemental Figure 2. Full Western Blot Images for Figure 4 E-G.

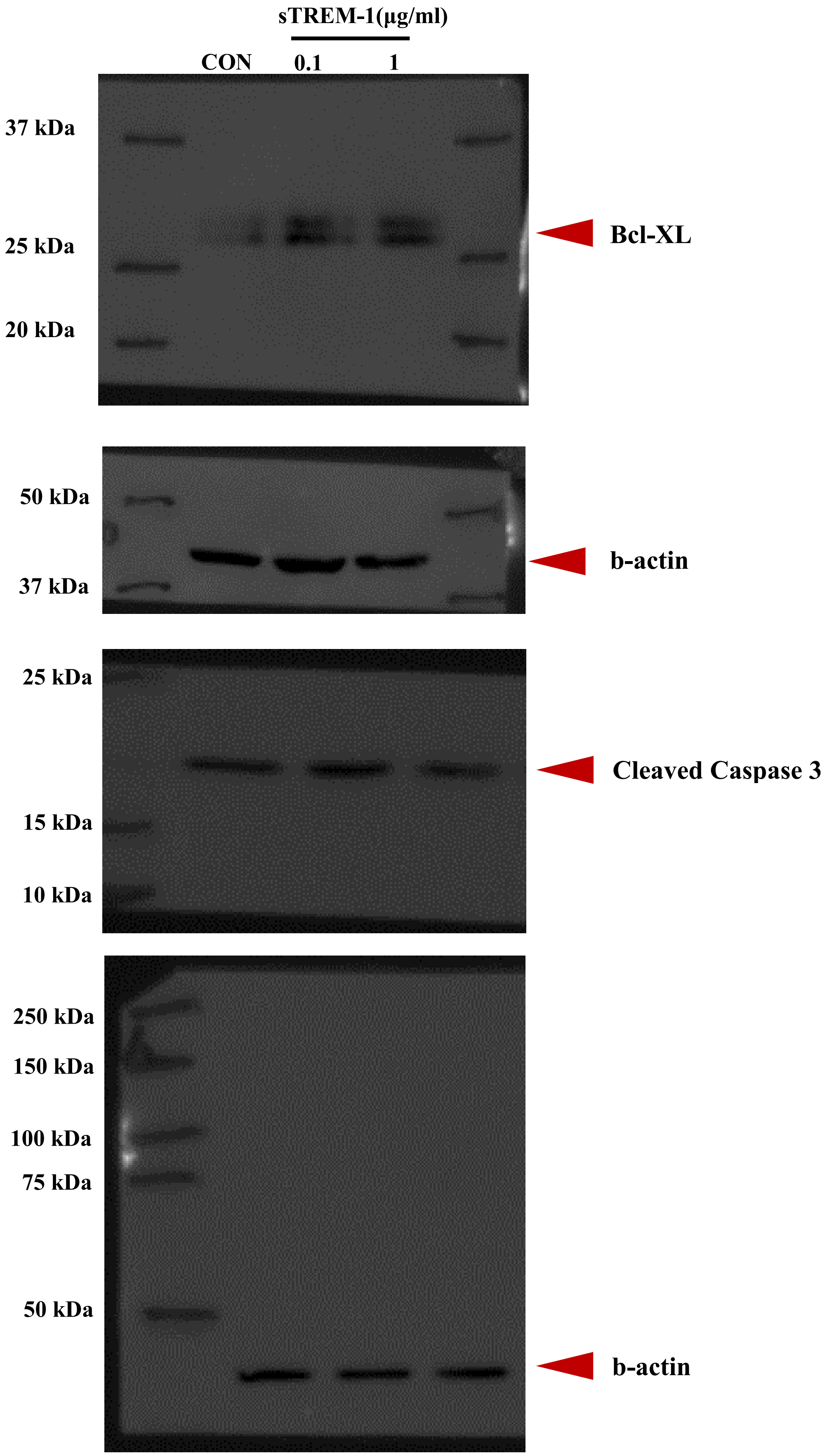

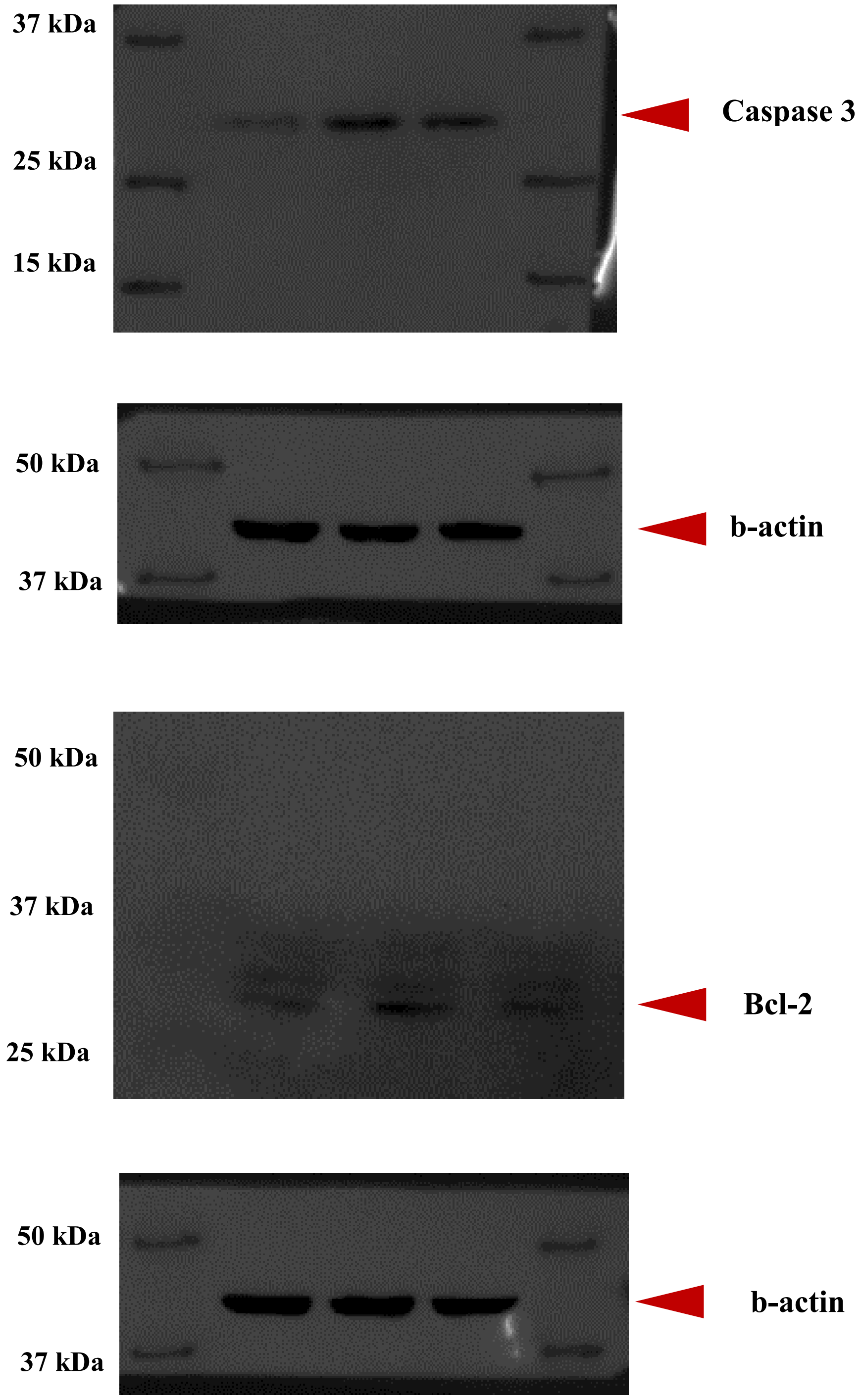
37 kDa
25 kDa
15 kDa
Caspase 3
50 kDa
37 kDa
b-actin
50 kDa
37 kDa
25 kDa
Bcl-2
50 kDa
37 kDa
b-actin

Supplemental Figure 3. Full Western Blot Images for Figure 5 F.

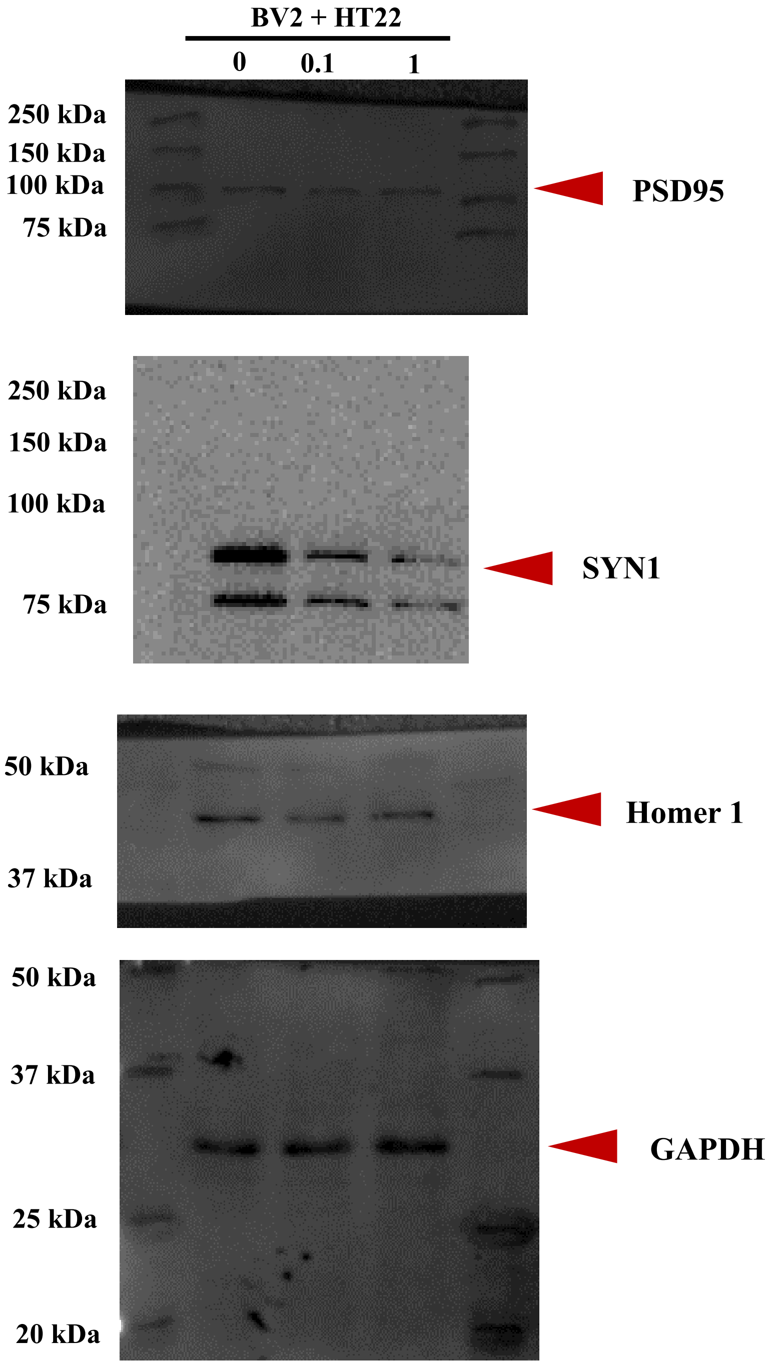

Supplemental Figure 4. Full Western Blot Images for Figure 6 B.

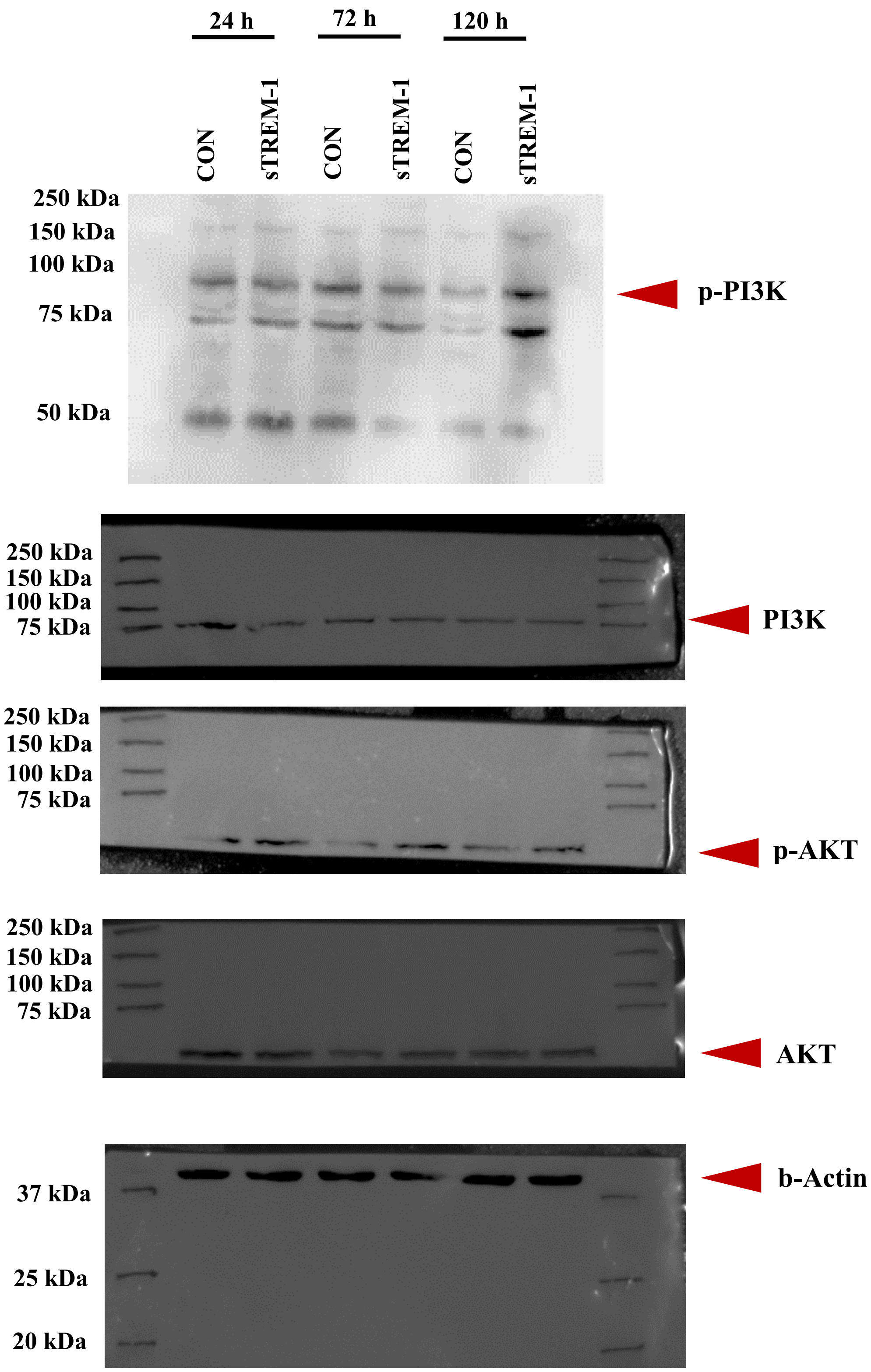

Supplemental Figure 5. Full Western Blot Images for Figure 6 E.

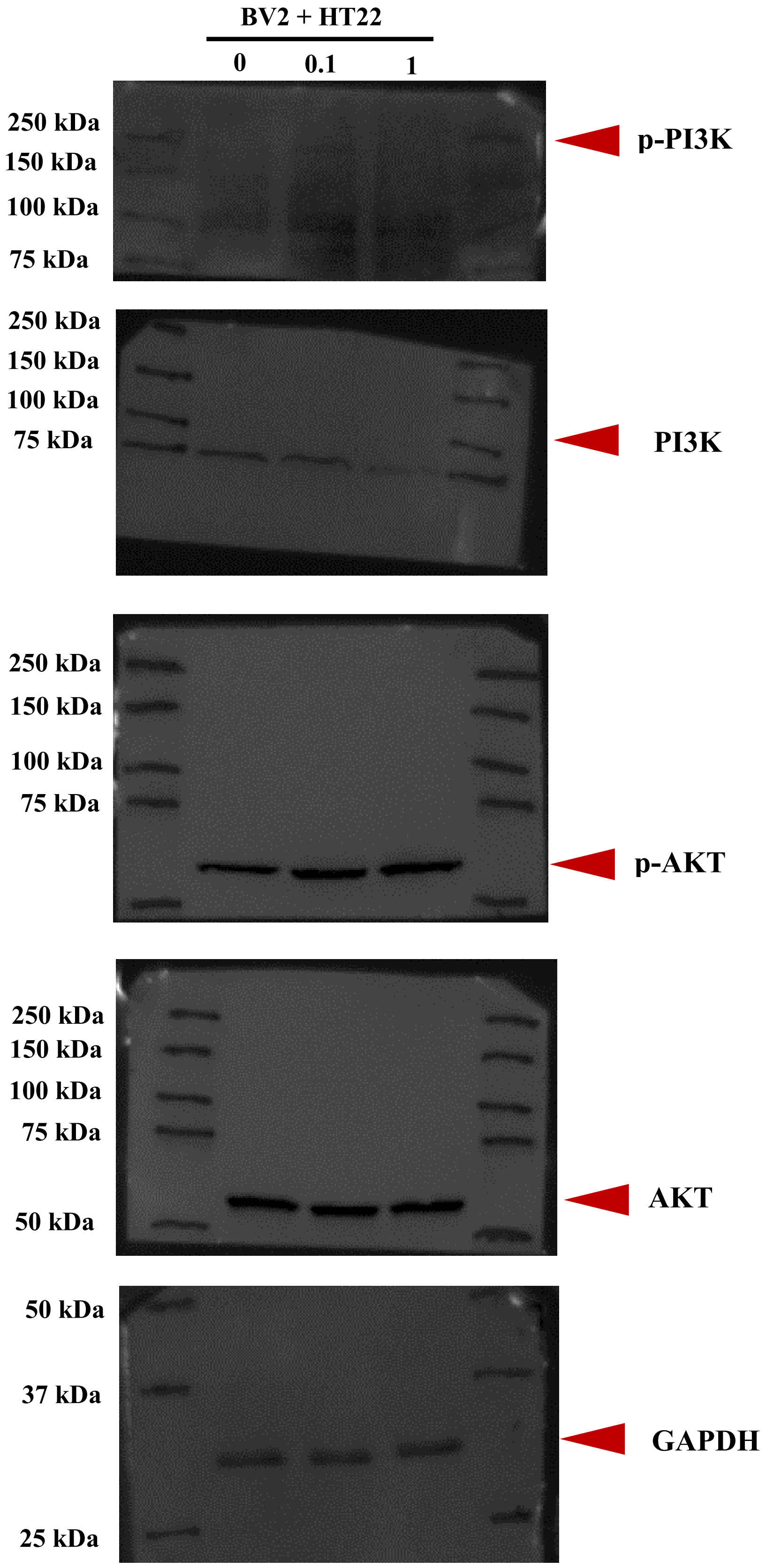

Supplemental Figure 6. Full Western Blot Images for Figure 7 C.

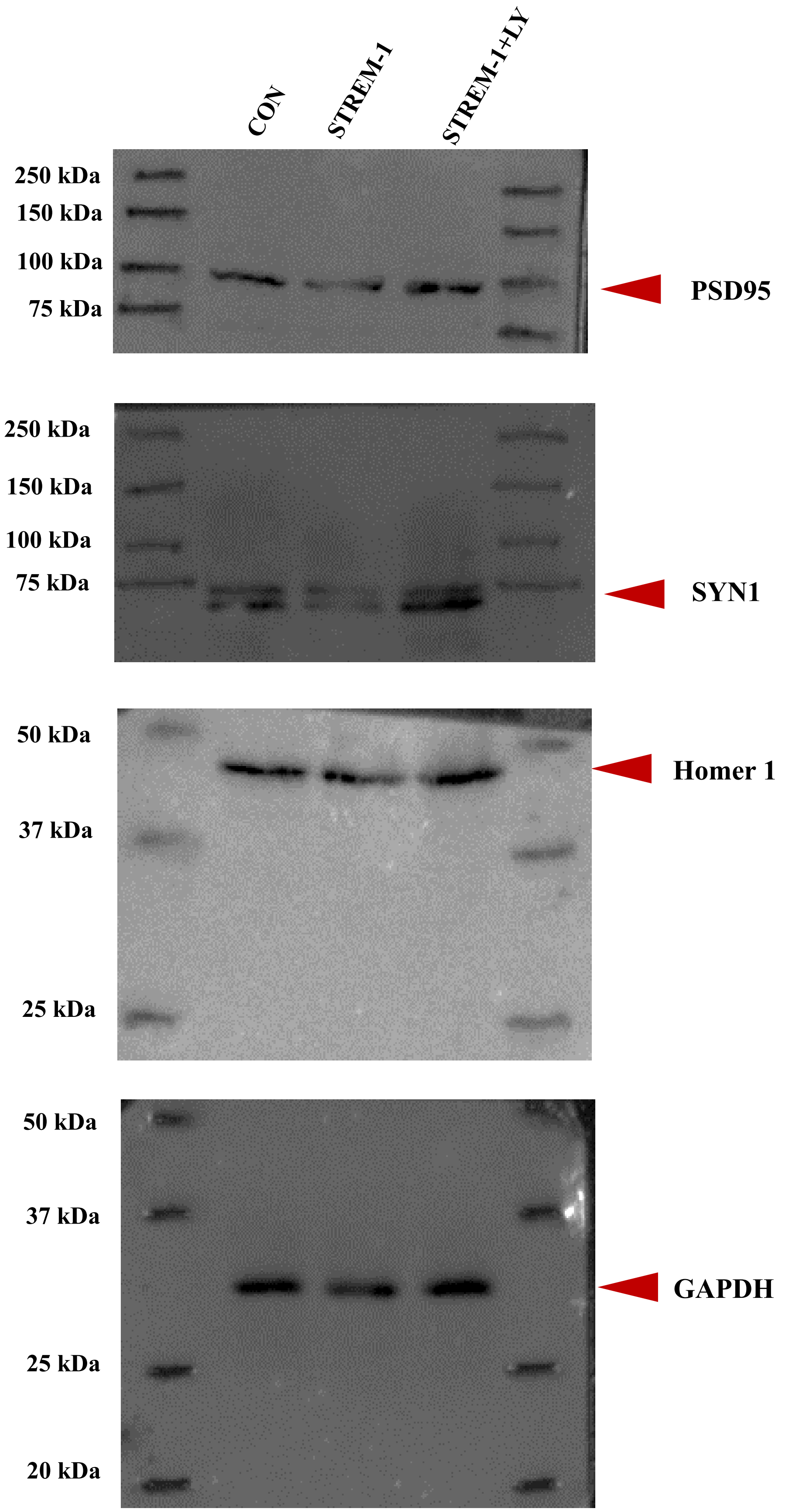

Supplemental Figure 7. Full Western Blot Images for Figure 7 I.

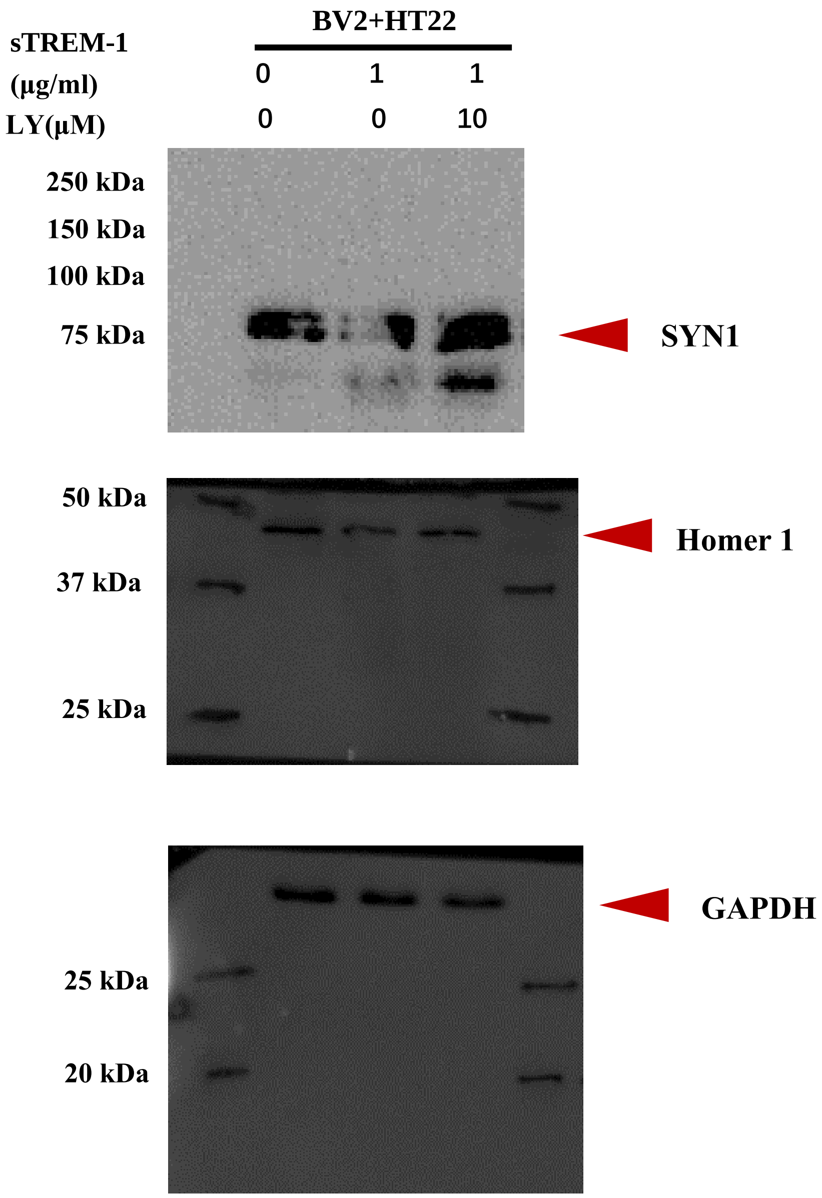

Supplemental Figure 8. Full Western Blot Images for Supplemental Figure 1 C.

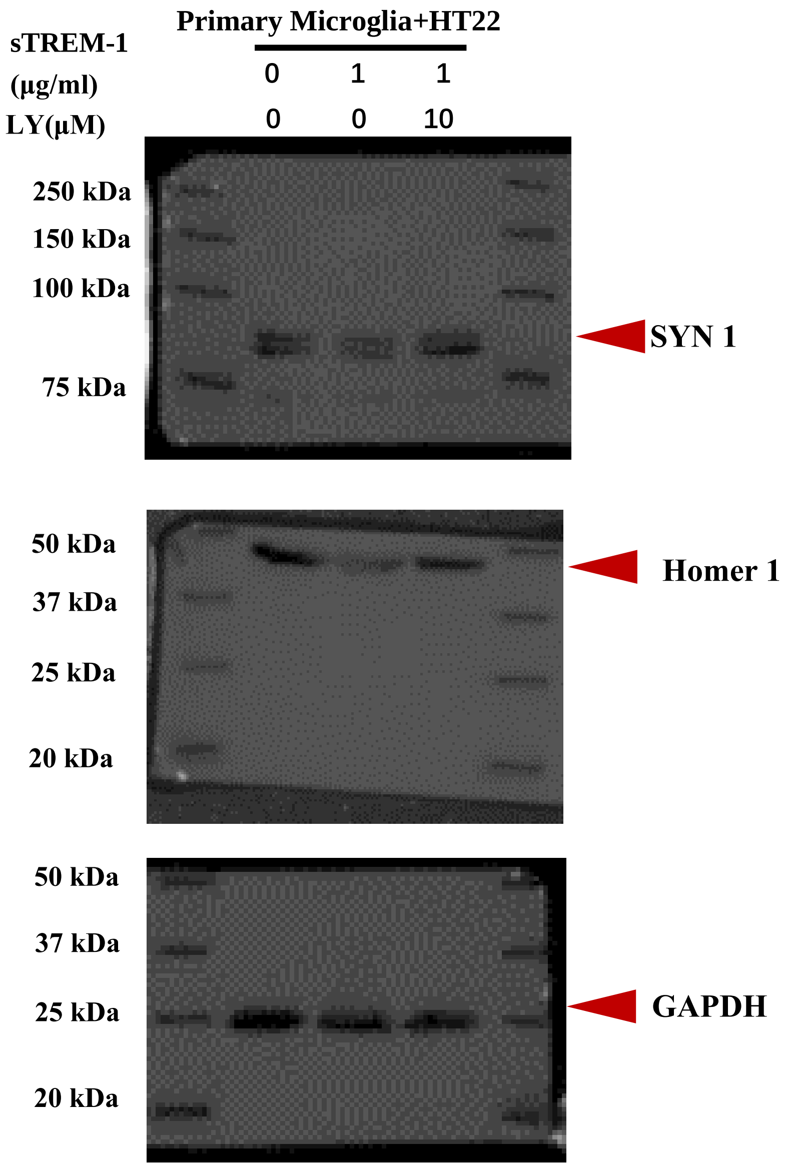

Supplemental Figure 9. Full Western Blot Images for Supplemental Figure 2 B.

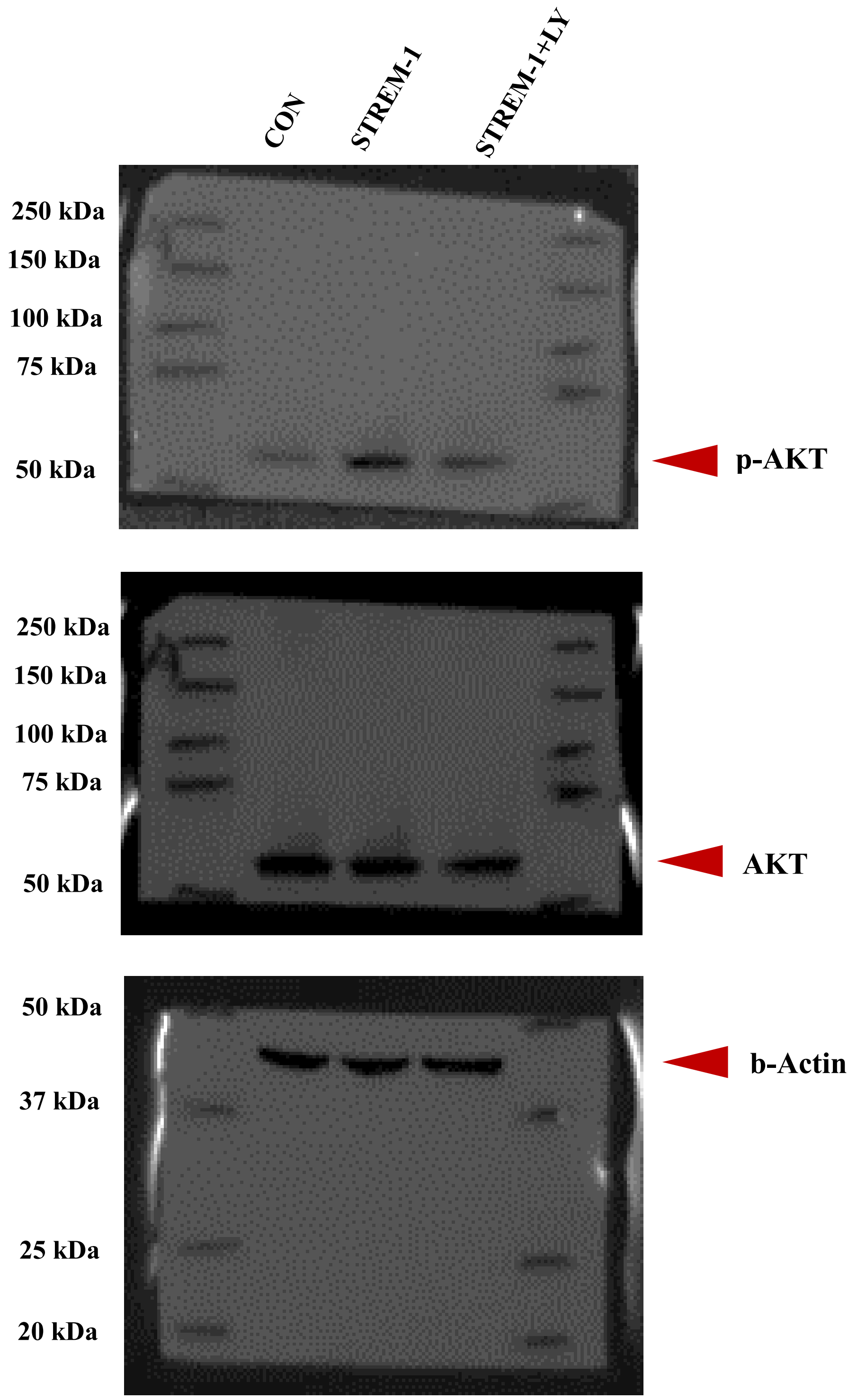

Supplemental Figure 10. Full Western Blot Images for Supplemental Figure 2 E.

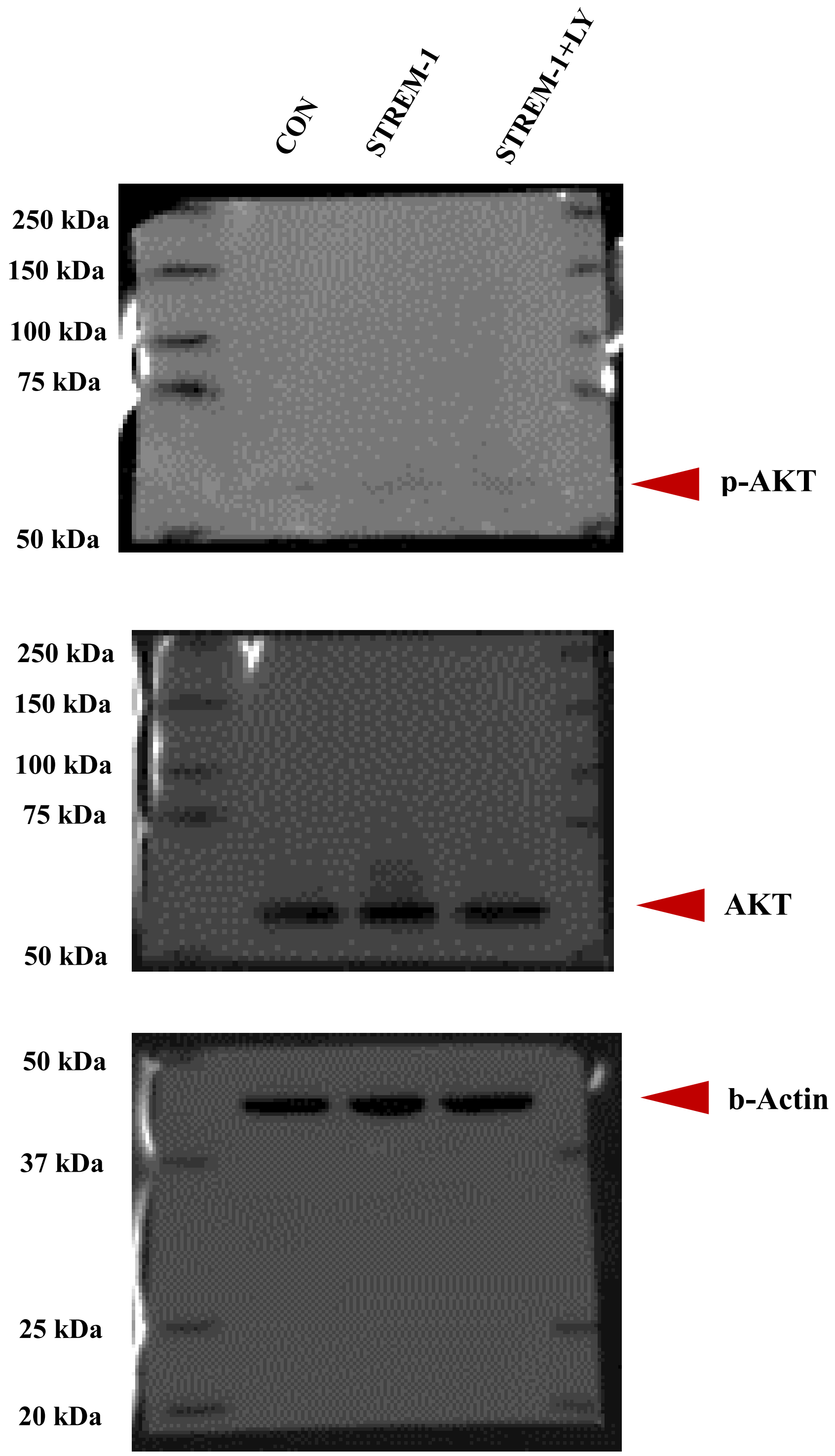

Supplement: Supplementary file 2 — Supplementary Figures. [file 41598_2022_10973_MOESM2_ESM.pdf]
